# Metabolite Quantitative Trait Loci Mapping for Fragrance and Fatty Acid Composition in Rice (*Oryza Sativa* L.)

**DOI:** 10.1186/s12284-025-00790-8

**Published:** 2025-10-22

**Authors:** Jeanaflor Crystal T. Concepcion, Mary J. Garson, Melissa A. Fitzgerald

**Affiliations:** 1 School of Agriculture and Food Sustainability, The University of Queensland, Brisbane, 4072 Australia; 2 School of Chemistry and Molecular Biosciences, The University of Queensland, Brisbane, 4072 Australia; 3 Carle R. Woese Institute for Genomic Biology, University of Illinois at Urbana-Champaign, Urbana, IL 61801 USA; 4 University of Al Dhaid, Al Dhaid, Sharjah, United Arab Emirates

**Keywords:** Metabolite QTL, Rice Fragrance, GC×GC-TOF-MS, GCMS, Oleic Acid, Linoleic Acid, 2-acetyl-1-pyrroline, Sulfur-containing Volatile Compound

## Abstract

**Background:**

Fragrance is a premium trait of grain quality in the traditional, Jasmine-style rice (*Oryza sativa* L.) variety Phka Rumduol (PRD). Rice fragrance is a combination of various volatile compounds with characteristic odours and flavours that can be traced back to fatty acids. Given the complexity of fragrance phenotype, the use of a robust phenotyping platform in identifying metabolite quantitative trait loci (mQTL) is essential to providing the most suitable set of genetic markers associated with desirable fragrance metabolites and their fatty acid origins in PRD-derived rice breeding lines.

**Results:**

Using combined untargeted metabolomics via GC×GC-TOF-MS, targeted fatty acid analysis with GC-MS, mQTL for individual volatile compounds and fatty acids were identified in a SNP-genotyped F_6_ recombinant-inbred rice population developed from a cross between PRD and Thmar Krem (TMK; non-fragrant). This study confirmed the genetic link between 2-acetyl-1-pyrroline and its derivatives with SNPs on chromosomes 8, 3, 4, and 1 and revealed novel mQTLs on chromosomes 2 and 6. MQTLs identified for saturated aldehydes, alcohols, ketones and alkylfurans co-localised on chromosomes 1, 3, and 6 were mainly contributed by PRD, whereas sulfur-containing compounds were contributed by TMK and associated with mQTLs on chromosomes 5 and 6. Oleic and linoleic acid composition were associated with a common QTL on chromosome 7.

**Conclusion:**

Our results demonstrate the ability of untargeted metabolomics to reveal the uniqueness of the fragrance profile of individual rice breeding lines in a mapping population which would allow for improved precision in marker-assisted selection. The various mQTLs verified in this study reaffirm the need for more in-depth metabolite-based selection incorporated in rice breeding. The information from this research is valuable for rice breeding and food quality improvement programs.

**Supplementary Information:**

The online version contains supplementary material available at 10.1186/s12284-025-00790-8.

## Background

Fragrance or aroma is valuable to the rice-consuming world, particularly because fragrant rice varieties command higher market prices and are a staple for many cultures (Fitzgerald et al. 2009; Mumm et al. 2016; Singh et al. 2000). One such culture is the nation of Cambodia, where rice is an essential part of the diet, comprising about 65–75% of daily energy consumption (Yu and Fan 2011). Fragrant rice is highly prized among Cambodian rice stakeholders, as shown by their preference for the traditional variety Phka Rumduol (PRD), an exemplary variety of fragrant rice (Concepcion et al. 2015, 2018). PRD is characterised by translucent, long slender grains that are fragrant and soft-textured after cooking (Concepcion et al. 2019), with quality traits acquired from years of traditional breeding. Since the early 1990s, about 2,000 rice (*Oryza sativa* L.) accessions have been collected, profiled, and stored in the gene banks of the International Rice Research Institute (IRRI) in the Philippines and Cambodia. However, only a few are widely grown in Cambodia due to agronomic adaptation, farmer adoption and consumer acceptance (Nesbitt 2003). Cambodian breeders have been actively selecting for the aromatic phenotype in PRD to combine aroma and yield (Vanyuth 2023); however, to use a variety as a breeding parent, its phenotype and genotype have to be known to facilitate the transfer of specific gene segments into new varieties (Concepcion et al. 2015).

Rice fragrance is widely measured by the concentration of 2-acetyl-1-pyrroline (2AP) in grains using gas chromatography-mass spectrometry (GC-MS) and by tasting cooked rice through sensory evaluation (Buttery et al. 1983; Daygon et al. 2016); each of these tests can only be achieved in late generations of breeding, when sufficient grain is available for milling (Concepcion et al. 2015). Sample availability therefore creates an obstacle to rapid selection, which in turn delays the identification of the fragrance phenotype. As a result, improved detection tools in the early stage of the breeding cycle such as genetic markers are most useful to breeders (Concepcion et al. 2015). The discovery and the genetic basis of 2AP have improved marker-assisted breeding for fragrance (Kovach et al. 2009; Shi et al. 2008). The fragrance phenotype via the presence of 2AP has been incorporated in breeding to develop high-quality rice that is tolerant to abiotic stress such as drought (Ndikuryayo et al. 2022) and with enhanced nutritional quality (Shi et al. 2023). However, fragrance is not associated with 2AP alone, as evidenced by the consistent detection and identification of other odour-active volatile compounds potentially derived from the oxidation of fatty acids in both fragrant and non-fragrant rice varieties (Calingacion et al. 2017; Concepcion et al. 2018; Daygon et al. 2016); which presents further complexity for breeders. Given the complexity of the fragrance phenotype, it is essential to have accurate and precise tools to identify the causative genes and proteins and to design robust markers that are linked to metabolites associated with a characteristic fragrance in a variety. Advancements in marker genotyping, accelerated by the development of single-nucleotide polymorphisms-based genotyping, has indeed made genotyping more readily available than ever before (McCouch et al. 2010). However, despite these advancements in marker technology and separation science, any genetic mapping efforts will not be successful without precisely acquired phenotypic data (Fitzgerald et al. 2009; Mumm et al. 2016; Singh et al. 2000). In the case of investigating the complex trait of rice fragrance, robust phenotyping techniques in separation science and mass spectrometry are incomparable to other methods of metabolite detection and identification (Calingacion et al. 2015; Mumm et al. 2016). This current study aims to investigate the genetic determinants of odour-active volatile compounds and fatty acids in a mapping population previously used for identifying quantitative trait loci (QTL) for rice textural quality (Concepcion et al. 2019). Data gathered here is generalised regarding the genetics and metabolites in Jasmine-style varieties using PRD as a representative of high-quality fragrant varieties.

## Methods

### Plant Materials and Genetic Data

The experimental materials consisted of 290 F_6_ recombinant-inbred lines (RIL) derived from the cross between two traditional *indicia* Cambodian rice varieties, PRD and TMK. These rice lines are a subset of the original 312 lines analysed for textural quality traits (Concepcion et al. 2019) selected based on sample availability. Seeds of the mapping population were grown in the experimental field of the Cambodian Agricultural Research and Development Institute (CARDI) and were managed under regular crop management procedures in the wet season of 2013. Plants were grown in a randomised complete block design with regular water and fertilizer management. Rice grains were harvested at physiological maturity. The harvested paddy from each variety was dehulled (Satake Rice Machine, Tokyo), milled (Grainman 60-230-60-2AT, Grain Machinery Mfg. Corp., Miami, FL), and a sub-sample was ground to flour (Udy Cyclone Sample Mill 3010–030, Fort Collins, CO) to pass through a 0.5-mm sieve. The molecular marker data of the PRD×TMK mapping population was obtained from the publicly available genome-wide SNP genotype data (Concepcion et al. 2019). This data set was acquired using Illumina’s *Infinium* 6 K SNP bead chip at the International Rice Research Institute’s Genotyping Services Lab.

### Untargeted Profiling of Volatile Compounds Using GC×GC-TOF-MS

Untargeted profiling of volatile compounds from milled rice flour (1 g) of 290 RILs, PRD, and TMK was carried out using a two-dimensional gas chromatography-time of flight-mass spectrometer (GC×GC-TOF-MS) (LECO, Australia) as described previously (Concepcion et al. 2018; Daygon et al. 2017). Quality control (QC) samples derived from pooled 0.2 g portion of each RIL were analysed after every 10 injections to cross-check the performance of the instrument and the consistency of runs across eleven batches of analyses (Beger et al. 2019). In addition, rice flour samples of Jasmine-type rice sourced from a local supermarket were also analysed, along with the experimental samples, to verify the presence of 2AP in fragrant rice lines. Low odour threshold analytical standards (Table S1) were run to confirm retention time and library annotation. Compounds with a signal-to-noise ratio (S/N) of at least 150 and a mass spectral library similarity of at least 80% were accepted (Table S2). Compounds that could not be confirmed using analytical standards or with mass spectra not aligning with the spectral data from Wiley Subscription Services, Inc. (US) mass spectral library were not included in post-data processing. Means of duplicate measurements were used for statistical analyses.

### Fatty Acid Analyses Using GC-MS

Lipids were extracted from rice flour of PRD, TMK and RILs according to methods described previously (Concepcion et al. 2018). Once total lipids were obtained, fatty acids in the oil extracts were derivatised into fatty acid methyl esters (FAMEs). Then, FAMEs were analysed using a GC-MS-QP2010 Ultra (Shimadzu Instruments, Singapore) with instrument settings described previously (Concepcion et al. 2018). The identity and area of the FAME peaks in the samples were confirmed both by matching the retention time (s) with the FAME standards using GCMS Solutions software (Shimadzu Scientific Instruments) and by matching the mass spectra to the NIST2011 library. Means of triplicate measurements were used for statistical analyses.

### Statistics

#### Volatile Compounds and Fatty Acids in the Grains

Compounds present in fewer than 50% of the QC samples, and column-derived compounds were excluded from the analysis. Descriptive statistics of all the phenotypic data as well as the generation of histograms were carried out using IBM SPSS Statistics 25 (IBM Analytics). Principal components analysis (PCA) and the generation of scores and loadings plots were carried out using SIMCA 14 (MKS Umetrics, Sweden). Fatty acid composition in the rice lipid extracts was expressed as the area percentage (%) of total fatty acids relative to the peak area and concentration of the internal standard (C17:0, 10 mg/mL) as described previously (Concepcion et al. 2018). Clustered heatmaps with Pearson’s *r* correlation values between the abundances of volatile compounds and between fatty acids were created in R version 4.3.1 and RStudio 2023.06.1 using the “cor” function within the “ggplot” package.

#### MQTL Identification

Metabolite QTL analyses were performed using Trait Analysis by Association, Evolution and Linkage (TASSEL; version 5.2.31) (Bradbury et al. 2007) and QGene 4.3.1 (Joehanes and Nelson 2008). Marker-trait association was assessed through a Generalised Linear Model (GLM) approach in TASSEL. In parallel, Composite Interval Mapping (CIM) was carried out on the data using QGene. The genetic distance between SNP markers was estimated from the physical map based on the Nipponbare genomic sequence available at GRAMENE (http://www.gramene.org), with genetic distance (cM) = Physical distance (kb)/260. CIM was done using a walk speed of 2 cM and cofactor selection of ‘auto’. A logarithm of odds (LOD) score of at least 3.0 was accepted and permutation tests with 1000 iterations at alpha = 0.05 (Churchill and Doerge 1994) were performed for each trait with GLM and CIM to validate the mQTLs. Once permutation tests were passed, candidate genes in the identified mQTL regions were identified from online databases: https://rice.uga.edu/ and Rice Gene Thresher (http://rice.kps.ku.ac.th/Site/index.html) and Phytozome v12.1 (https://phytozome.jgi.doe.gov/pz/portal.html#).

## Results

### Profiling of Volatile Compounds in the Rice Mapping Population

**Fig. 1:**
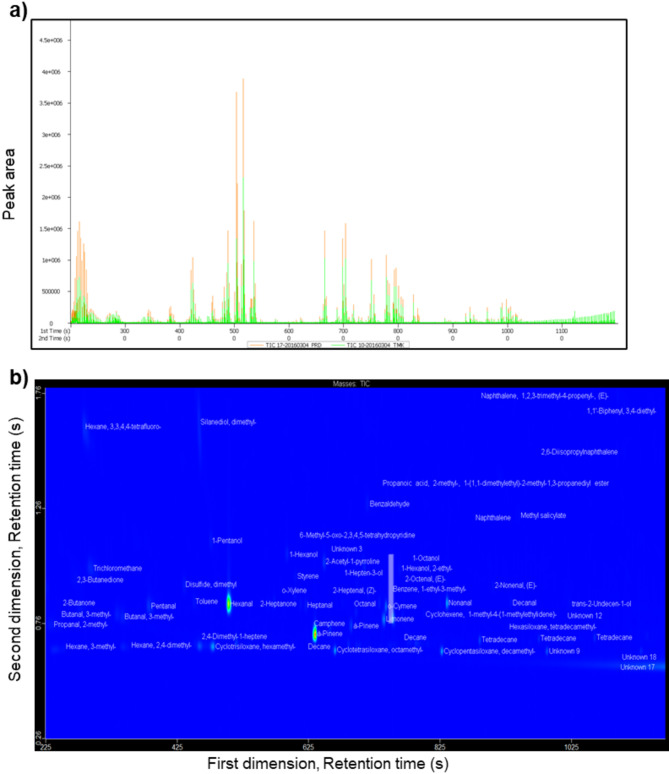
**a** Representative total ion chromatogram of volatile organic compounds in milled rice samples of fragrant rice variety Phka Rumduol (PRD) and non-fragrant rice variety Thmar Krem (TMK). **b** Sample contour plot of volatile compounds from pooled samples of 290 F6 recombinant-inbred lines derived from the cross between PRD and TMK detected via two-dimensional gas chromatography time-of-flight mass spectrometry

**Fig. 2:**
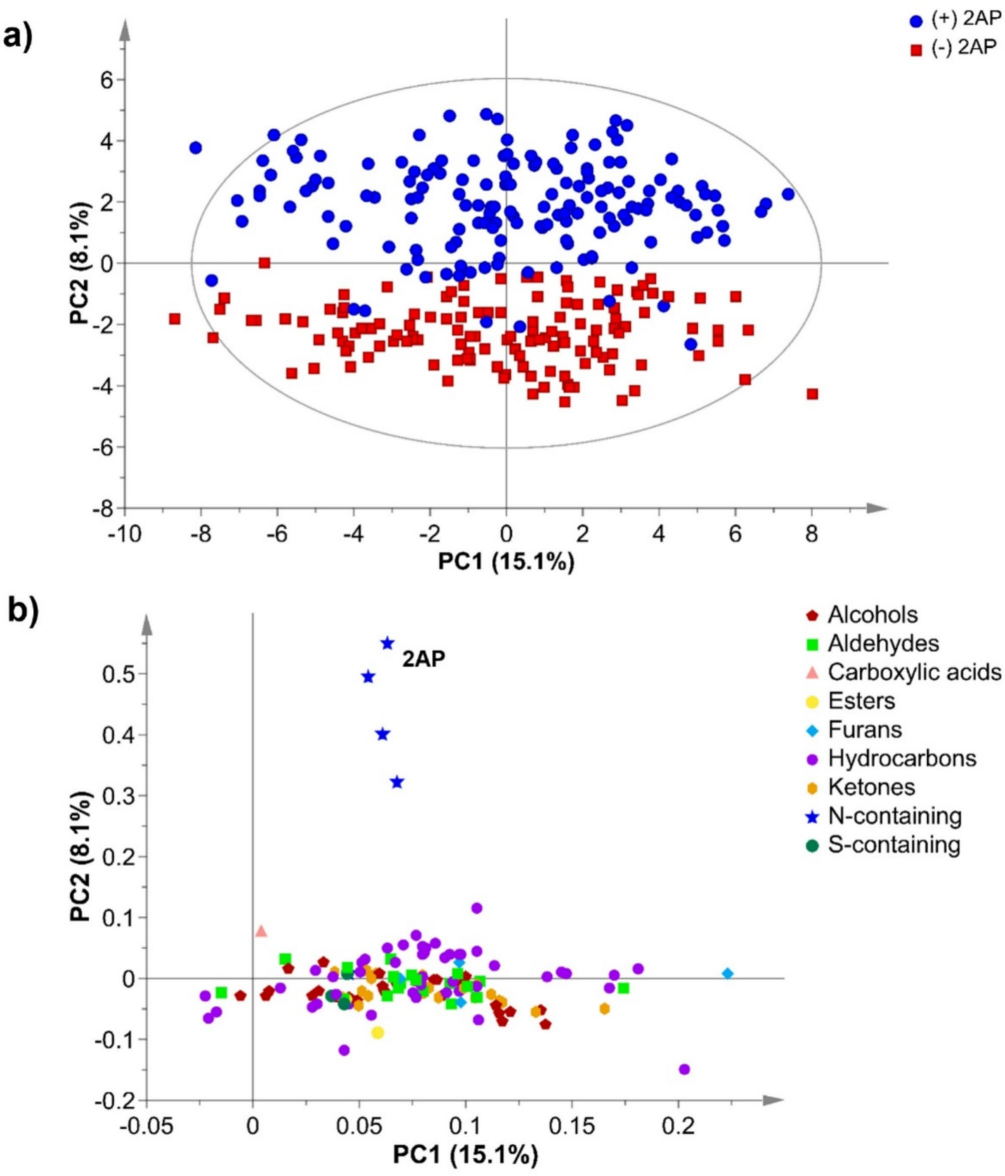
Principal components analysis **(a)** scores scatter plot of 290 F_6_ recombinant-inbred lines derived from the cross between Phka Rumduol (PRD) and Thmar Krem (TMK) and **(b)** the loadings plot of 131 volatile compounds detected using two-dimensional gas chromatography-time of flight-mass spectrometry

The total ion chromatogram of the parental rice lines PRD and TMK indicated differences in peak abundance and volatile profile between these two varieties (Fig. 1a). The RIL population produced volatile compounds separated on the primary and secondary columns of the GC×GC-TOF-MS and annotated using an in-house library (Fig. 1b). A total of 131 volatile compounds including hydrocarbons, aldehydes, alcohols, ketones, esters, N-containing compounds and S-containing compounds, furans, and carboxylic acids were detected in rice grains of 290 RILs (Fig. 2). Fragrant rice lines with 2AP (blue), were separated from the non-fragrant lines (red) (Fig. 2a). Of these 290 RILs, 124 do not have 2AP (peak area = 0), whereas 16 have peak areas from 2533 to 94,000, 52 from 10,000 to 94,000, 65 with areas from 101,965 to 193,575 and 33 with values from 200,533 to 537,367. This separation along PC2 was largely due to 2AP and its associated N-containing volatile compounds. The variation in PC1 (15.1%) however, was contributed by other volatile compounds such as odour-active aldehydes, alcohols, ketones and hydrocarbons that are known to be derived from further degradation of lipids (Fig. 2b).

**Table 1:** Volatile compounds, their odour threshold and description, and relevant statistics detected in the headspace of milled rice mapping population derived from the cross between PRD and TMK

Compound	Odour description ^c^	Odour threshold (ppb) ^d^	RIL (*n* = 290)						
			Mean	SD	Min	Median	Max	Skewness	Kurtosis
***N*** **-** **containing**									
2-Acetyl-1-pyrroline ^adefghijn^	popcorn-like	0.1 ^j^	73341.30	98146.20	0.00	14145.30	537367.00	1.50	2.50
6-Methyl-5-oxo-2,3,4,5-tetrahydropyridine ^aln^	popcorn		4155.70	6635.00	0.00	0.00	41377.10	2.30	6.20
2-Acetylpyrrole ^adfln^	nutty	170,000	1078.40	2577.00	0.00	0.00	25143.90	4.60	32.40
Pyrrole ^afkn^	nutty, roasted	^f^	3735.00	3368.00	0.00	3444.00	14308.00	0.44	-0.69
**Aldehydes**									
Butanal ^adf^	chocolate	9	363955.40	132858.60	90981.60	338340.50	872974.00	0.90	0.90
Pentanal ^adfghjm^	fermented	12	215011.90	103166.60	77253.60	189468.00	941405.00	2.70	11.90
Hexanal ^adefghijkm^	green	4.5 ^j^	1879699.00	1025560.80	699369.00	1633680.00	9317620.00	2.90	13.20
Heptanal ^adefhijm^	green	3	88097.40	26236.80	34648.80	84075.10	201115.00	0.90	1.40
Octanal ^adefhijm^	aldehydic	0.7	70577.50	18870.90	32973.60	68137.10	170523.30	1.20	3.60
Nonanal ^adefghjkm^	fruity or floral	1	273022.80	69277.40	136779.50	265048.50	626195.00	0.90	2.20
Decanal ^bdehijm^	aldehydic, soapy	0.1 ^j^	64160.50	16896.10	32613.30	62724.30	135031.00	0.70	0.90
Dodecanal ^b^	aldehydic; soapy	2 ^d2^	9247.50	6127.80	2430.70	7640.00	40067.60	2.50	7.00
Benzaldehyde ^bdefhjm^	bitter almond	350 ^j^	95197.20	30976.00	36072.10	91522.10	223447.00	0.80	1.10
2*Z*-Heptenal ^adehiljm^	green ^e^	13	19646.50	9816.00	3509.30	17194.90	79402.40	2.20	8.70
2*E*-Hexenal ^behm^	green	17	2715.00	1007.30	967.30	2577.10	11519.50	3.20	21.50
2*E*-Nonenal ^bdehjm^	fatty	0.1 ^j^	14827.20	6059.50	2466.90	15273.60	33604.70	0.30	-0.50
2*E*-Octenal ^befhjmm^	fatty	3 ^j^	7441.10	3413.80	1469.10	7238.10	21962.00	1.10	1.60
2*E*,4*E*-Nonadienal ^beh^	green, fatty		3062.40	1913.10	0.00	3259.10	12418.20	0.20	1.10
2-Methyl butanal ^bfk^	burnt	1 ^d1^	519460.50	281377.60	0.00	518053.00	1513220.00	0.50	0.80
2-Methyl propanal ^bn^	aldehydic, pungent	0.9 ^d2^	1042629.30	425737.10	295809.00	958033.50	3324630.00	1.30	3.10
2-Ethyl hexanal ^bi^	mild odour		23174.10	8770.10	5002.20	22384.10	58116.20	0.50	1.00
3-Methyl butanal ^bfm^	ethereal	0.15 ^d2^	713458.10	419746.10	0.00	602861.00	2823310.00	2.00	5.40
**Alcohols**									
1-Butanol ^bdmkn^	fermented	500	910749.30	323614.80	310027.00	836142.50	2501610.00	1.60	4.00
1-Heptanol ^aeghmn^	green ^c^	200 ^d2^	14403.00	5208.40	3357.20	14016.40	33362.40	0.50	0.50
1-Hexanol ^bdefghijkmn^	herbal, grassy ^j^	2500 ^j^	272191.50	123097.10	99550.30	240986.50	1115990.00	2.00	7.70
1-Octanol ^bdehjmn^	waxy	110 ^j^	21899.10	6432.10	11959.80	20517.10	51207.40	1.20	2.20
1-Octen-3-ol ^bdefhjmn^	earthy	1 ^j^	155367.90	64780.20	74591.10	141201.50	553522.00	2.40	9.20
1-Pentanol ^adefhmn^	fermented	4000	160195.90	63672.20	79420.50	144280.00	456090.00	1.90	4.40
1-Penten-3-ol ^bkn^	green, hay-like ^k^	400	52825.90	23642.10	0.00	50557.90	193922.00	1.20	5.30
1-Propanol ^bn^	mild, alcohol-like		149477.50	109324.70	7286.30	137346.00	1068420.00	3.10	19.90
2-Methyl-1-propanol ^bjn^	ethereal, winey	7000 ^j^	181143.30	66677.80	24482.80	173232.00	468986.00	0.60	1.80
2-Butanol ^bn^	fruity		242970.10	125525.10	0.00	225493.30	903689.00	1.50	4.50
2-Hexanol ^ben^	winey	2500	32261.90	32547.60	0.00	17987.80	238124.50	2.30	8.60
2*Z*-Octen-1-ol ^bn^	fatty		3748.60	3088.20	0.00	3080.70	25150.00	3.80	19.50
2-Pentanol ^be^	fermented		78888.10	73313.70	6118.20	79085.90	497623.50	1.70	5.40
2-Butyl-1-octanol ^bmn^			49575.20	20335.20	0.00	45652.30	144146.10	0.70	1.10
2-Ethyl-1-hexanol ^bjmn^	floral, green	270,000 ^j^	190125.00	70125.70	76088.10	180121.50	510811.00	1.30	2.50
2-Methyl-2-propanol ^bn^	ethereal winey	7000 ^j^	189457.20	243150.30	0.00	101466.50	1511720.00	2.70	8.90
3-Methyl-1-butanol ^bdj^	fermented, malty	250	79839.00	29995.90	11139.50	73809.90	195856.00	1.10	1.40
Phenol ^bi^			14337.80	10647.40	5527.80	13517.30	189112.00	15.40	253.50
**Ketones**									
2-Butanone ^bj^	fruity, ethereal	50,000 ^j^	1610608.10	667083.70	165444.00	1542530.00	3923760.00	0.60	0.70
2-Decanone ^beh^	floral		4783.10	1996.40	0.00	4265.00	14313.30	1.70	3.50
2-Heptanone ^adefhm^	cheesy	140	113735.60	57409.30	47123.60	97051.90	464603.00	2.70	10.00
2-Hexanone ^bn^	fruity		28063.80	20592.30	8881.50	20261.00	122801.00	2.20	5.20
2-Nonanone ^bdeh^	fruity	200	5067.30	2170.30	0.00	4889.40	17760.50	1.10	5.20
2-Octanone ^ad^	earthy, dairy	28	11942.50	3704.80	5769.80	11190.80	29746.00	1.50	3.10
2-Pentanone ^bn^	fruity	2300 ^d6^	64206.30	37410.30	11546.90	63214.30	221822.00	1.10	2.00
2-Undecanone ^ad^	fruity	7	4920.20	4125.10	0.00	3222.60	27050.50	2.00	5.60
3-Octanone ^bd^	herbal	28	3380.80	1468.20	912.60	3074.20	11698.90	1.70	5.90
3-Octen-2-one ^bej^	earthy		6607.20	4022.20	0.00	5539.60	27856.20	2.40	6.80
3-Penten-2-one ^bcm^	fruity, fishy	1.5	18129.60	37148.80	0.00	6009.70	315476.00	3.60	17.90
2,3-Butanedione ^akn^	creamy, buttery	3	296118.90	227205.50	13867.40	256554.00	1879980.00	2.50	12.10
Acetoin ^afjkn^	dairy, butter ^j^	600–750	2508.20	8602.00	0.00	0.00	128583.00	11.20	160.60
Acetophenone ^bdeikn^	floral	65 ^j^	44374.70	30611.00	4421.70	31154.20	128424.00	0.70	-0.80
Cyclopentanone ^bm^			4199.00	2203.00	0.00	3837.90	21559.80	2.10	13.10
4-Hydroxy-4-methyl-2-pentanone ^bin^			27246.70	52693.50	0.00	16560.60	722188.00	8.80	106.80
Benzophenone ^bi^	herbal		7478.20	18852.80	0.00	0.00	143826.00	3.90	17.40
**Furans**									
2-*n*-Butylfuran ^bgin^	fruity		9351.10	6694.60	1536.60	8663.20	54851.80	2.70	12.50
2-Ethylfuran ^bkn^	powerful, sweet, burnt	6	3109.40	3073.40	0.00	2770.20	28708.20	6.30	46.00
2-Pentylfuran ^adefhijmn^	beany	6	103285.40	42342.10	45406.70	92269.90	329880.00	2.00	6.00
**S-containing**									
Benzothiole ^bh^	sulfurous	80 ^d3^	6574.00	191.00	1730.20	5316.90	16003.60	1.00	0.10
Dimethyl disulfide ^bn^	sulfurous	12 ^d5^	243999.10	63966.50	84613.10	239758.00	607123.00	0.80	2.90
Dimethyl trisulfide ^afn^	alliaceous	0.01	12697.30	6660.50	1750.10	11368.50	50249.70	1.40	4.10
**Hydrocarbons**									
3,7-Dimethyl-1-octene ^bn^	woody		99506.60	92167.40	0.00	64780.90	578050.00	2.20	5.50
1-Decene ^b^			55117.97	106622.69	0.00	24613.05	818435.00	3.81	16.23
3*Z*-Dodecene ^bm^			73869.80	187898.50	0.00	35910.10	1619170.00	5.70	34.00
3*Z*-Tridecene ^bm^			3531.20	5288.60	0.00	3254.10	51870.30	5.40	41.90
Propyl benzene ^bin^	floral		45228.10	29101.40	9459.60	35190.60	191572.00	2.10	5.30
Ethyl benzene ^b^			226591.00	157253.60	25219.50	178095.00	1350960.00	2.00	8.40
Hexadecane ^bmn^			59547.70	18582.40	16291.70	56695.80	129856.00	1.50	3.20
Camphene ^bin^	camphor		5942.90	5167.60	0.00	3442.40	26037.90	1.60	1.40
Aromadendrene ^bn^	woody		85197.60	61310	0.00	77092.90	267374.00	0.50	-0.80
Limonene ^adefikmn^	citrus	60 ^d4^	721.90	838.10	0.00	0.00	3732.10	0.70	-0.60
o-Cymene ^bin^			7443.30	6387.20	0.00	5827.20	69368.20	4.10	31.50
α-Phellandrene ^bin^	terpenic		486.00	902.40	0.00	0.00	5671.40	2.40	7.70
α-Pinene ^bikn^	herbal	9.5 ^d4^	72410.60	43729.40	14755.90	54072.60	311995.00	1.60	3.30
α-Methylstyrene ^bi^			6545.40	4456.90	1874.40	3906.20	17396.00	0.80	-1.00
Heptane ^an^			338007.40	142194.10	54143.30	316554.50	790346.00	0.70	0.40
Octane ^an^			597605.70	289195.40	122669.00	546654.00	1915780.00	1.60	4.40
Tetradecane ^aeimn^	mild waxy		1106166.00	1127694.00	339189.00	696647.50	5628560.00	2.60	5.90
Tridecane ^amn^	hydrocarbon		345751.30	186186.90	61646.50	336584.50	1060945.00	0.50	-0.20
Undecane ^amn^			1809117.00	1081118.00	321803.00	1683020.00	9022780.00	1.90	7.80
Dodecane ^bmn^			2790308.00	1622346.00	1012900.00	2181550.00	9504860.00	1.80	2.90
p-Xylene ^bin^	sweet		442681.80	346205.10	48063.80	340947.50	3761390.00	3.70	28.80
Benzene ^bn^			87007.60	42369.80	10996.30	76486.70	227682.00	1.00	1.10
Toluene ^bn^			1394185.00	593711.00	165969.00	1326780.00	2944710.00	0.40	-0.60
2-Methyl naphthalene ^bh^	naphthalene	2.5 ^n^	3663.90	1858.40	1516.30	3245.40	15321.70	3.00	11.40
1,2,3-Trimethyl benzene ^bn^			190379.80	143258.50	64600.40	151371.80	1500660.00	4.50	29.80

^a^ Compound identity confirmed by comparison with mass spectra and retention time of analytical standards; ^b^ Compound putatively identified; mass spectrum agrees with spectrum in Wiley Subscription Services, Inc. (US) mass spectral library; ^c^ Odour description derived from the Good Scents Company website (http://www.thegoodscentscompany.com) unless otherwise specified; ^d^ Odour threshold derived from Buttery et al. (1988) unless otherwise specified: ^d1^ Moore et al. (1976); ^d2^ Guadagni et al. (1963); ^d3^ Takeoka et al.undefined (1990); ^d4^ (Ahmed et al. 1978); ^d5^ Hansen et al.undefined (1992). These compounds were previously reported in rice: ^d^ Buttery et al. (1988); ^e^ Widjaja et al.undefined (1996); ^f^ Buttery et al. (1999); ^g^ (Calingacion et al. undefined^2012^); ^h^ Yang et al.undefined (2008); ^i^ Calingacion et al. (2015); ^j^ Griglione et al. (2015); ^k^ Daygon et al. (2016); ^l^ Daygon et al. (2017); ^m^ Calingacion et al. (2017); ^n^ Concepcion et al. (2018)

### N-Containing Compounds

Five N-containing volatile compounds were identified in this study, four of which have previously been reported in rice (Table 1). These compounds include the cyclic odour-active volatile compounds 2AP, 2-acetylpyrrole, 6-methyl-5-oxo-2,3,4,5-tetrahydropyridine (6M5OTP) and pyrrole.

### Furans

Among the three furans detected in the rice samples (Table 1), 2-pentylfuran and 2-*n*-butylfuran, have been previously reported in rice and are known products of linoleic acid and oleic acid oxidation, respectively (Frankel 1983). The identity of 2-pentylfuran was confirmed using an analytical standard (Table S1).

### S-Containing Compounds

Three odour-active S-containing volatile compounds were detected and putatively identified in the population. These compounds include dimethyl disulfide (DMDS), dimethyl trisulfide (DMTS), and benzothiole (Table 1).

### Aldehydes, Alcohols, Ketones, and Hydrocarbons

Eighteen aldehydes, 18 alcohols, 17 ketones and 25 hydrocarbons were identified in the rice samples. Of these, eight were saturated aldehydes that ranged from butanal (C4) to dodecanal (C12), five unsaturated aldehydes, one cyclic and four alkylated aldehydes. The alkylated aldehydes that were putatively identified and are known to have undesirable odours include 2-ethyl hexanal which is associated with sharp and powerful odour, 2-methyl butanal, 2-methyl propanal and 3-methyl butanal. Several hydrocarbons were putatively identified, although most of them do not have low odour thresholds (Table 1). Compounds not related to lipid oxidation included terpenes.

### Correlations Between Rice Volatile Compounds

**Fig. 3:**
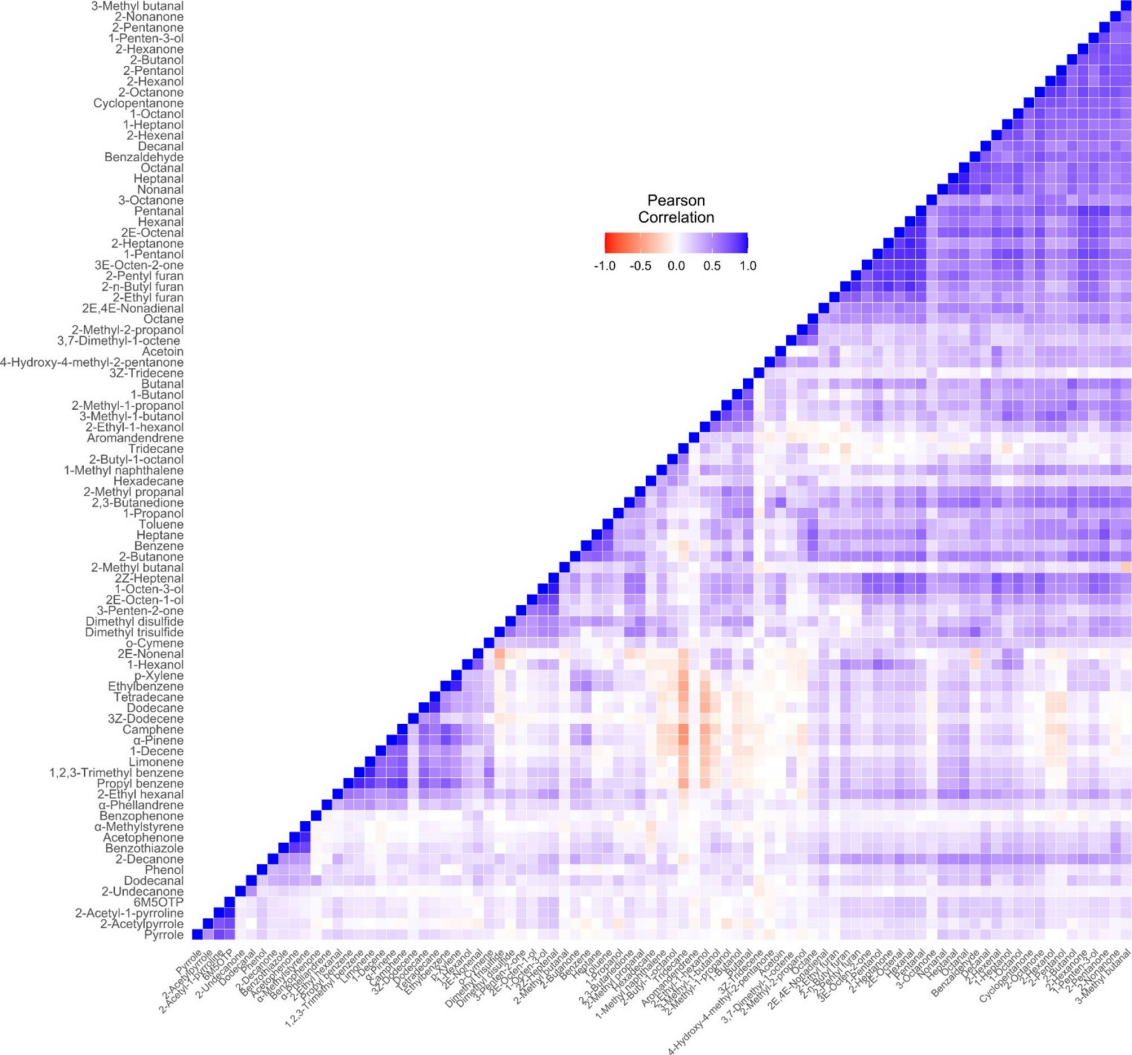
Correlation heatmap showing 88 volatile compounds and their correlation coefficient score represented by coloured boxes

A correlation heatmap was generated to further visualise the relationships between the odour-active volatile compounds detected in the RIL population (Fig. 3).  . Correlation values indicate 2AP exhibiting strong positive correlations with its derivatives pyrrole (*r* = 0.60), 6M5OTP (*r* = 0.85), and 2-acetylpyrrole (*r* = 0.57). Hexanal, the main marker for fatty acid oxidation, was identified as positively strongly correlated with other volatile compounds known to be derived from rice unsaturated fatty acid oxidation, and these compounds include the following: 2–heptanone (*r* = 0.93), 1-heptanol (*r* = 0.50), 1-hexanol (*r* = 0.35), 1-octanol (*r* = 0.64), 1-octen-3-ol (*r* = 0.71), 1-pentanol (*r* = 0.88), 1-penten-3-ol (*r* = 0.52), 1-propanol (*r* = 0.38), 2*E*,4*E*-nonadienal (0.39), 2*E*-heptenal (*r* = 0.76), 2-hexenal (0.69), 3-octen-2-one (*r* = 0.52), pentanal (0.94), 2*E*-octenal (*r* = 0.88), 2-pentylfuran (*r* = 0.874), and 2-*n*-butylfuran (*r* = 0.89) (Fig. 3). These alkylfurans were also strongly correlated with each other. DMDS and DMTS were also strongly positively correlated with each other (*r* = 0.70).

### Fatty Acid Profile of the Mapping Population

**Fig. 4:**
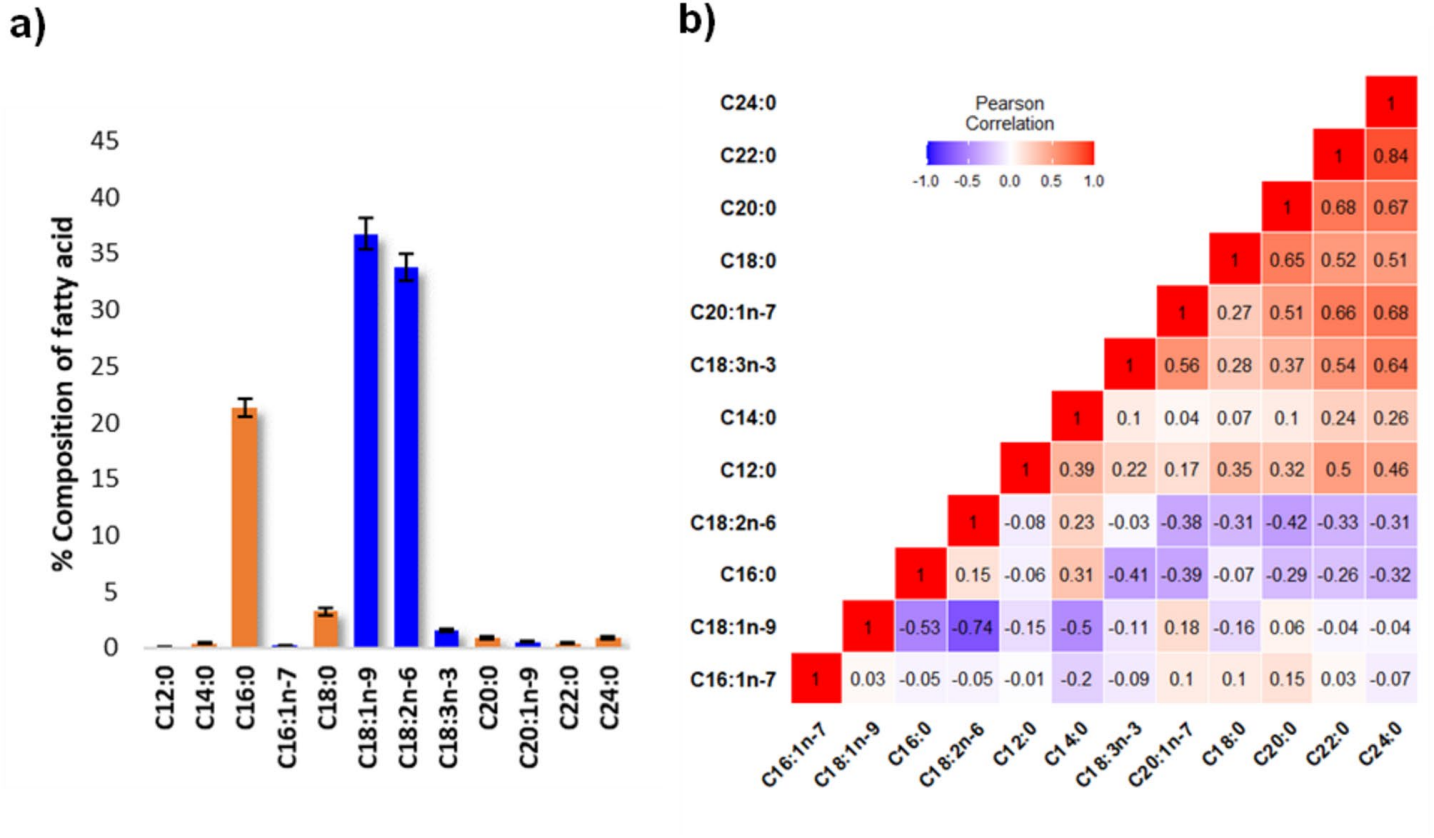
Fatty acids identified in the 210 F_6_ recombinant-inbred lines (RIL) derived from the cross between Cambodian rice varieties Phka Rumduol and Thmar Krem rice varieties harvested during the wet season of 2013 at the Cambodian Agricultural Research and Development Institute. **(a)** Mean percentage composition and **(b)** Pearson’s correlation coefficient (*r*) heatmap of % composition of 12 fatty acids identified in the RIL population. Orange bar graphs in **(a)** refer to saturated fatty acids whereas blue bar graphs refer to unsaturated fatty acids

**Fig. 5:**
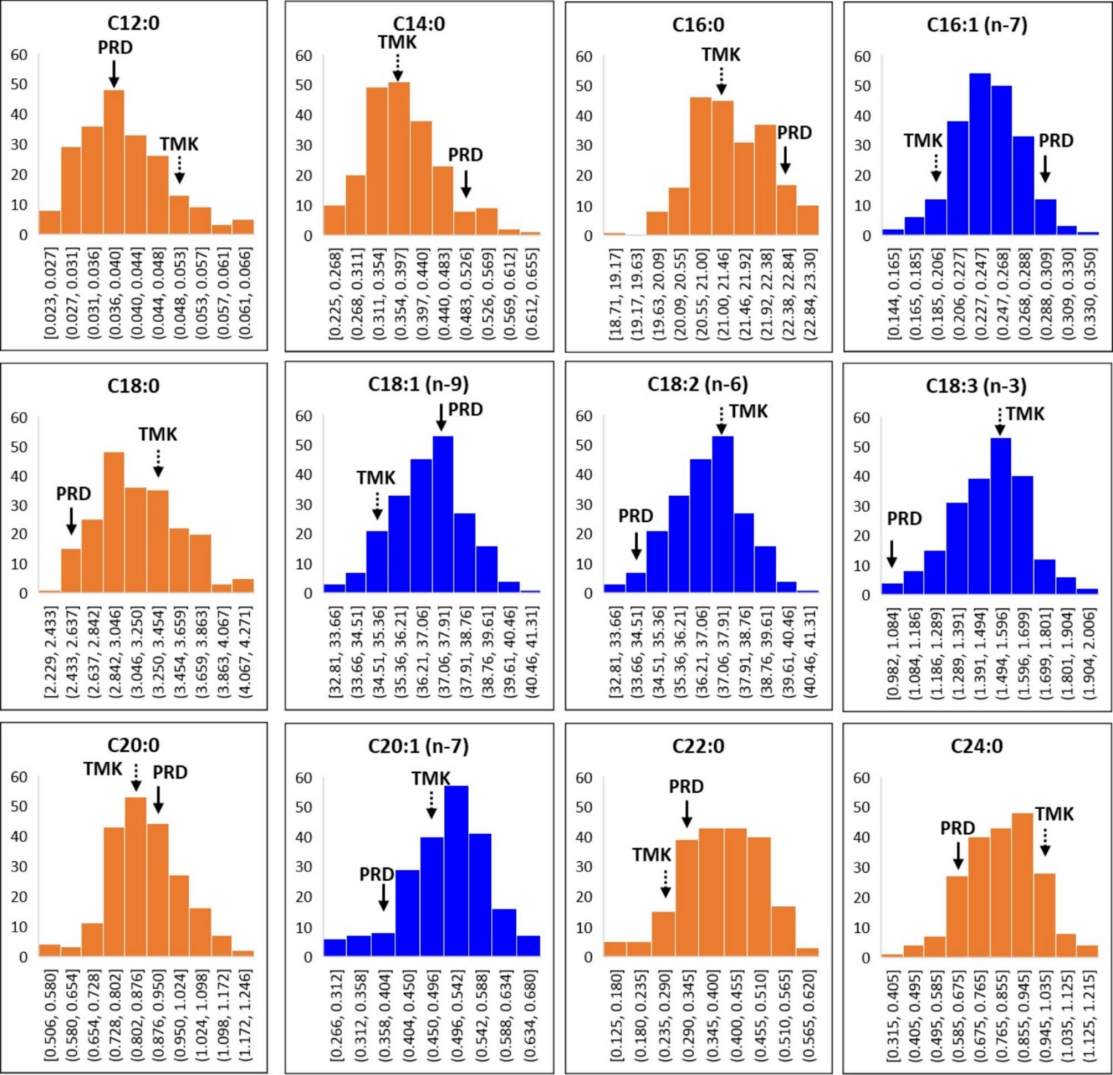
Frequency distributions of 12 fatty acids based on % composition in rice grains of 210 F_6_ recombinant-inbred lines derived from the cross between Cambodian rice varieties Phka Rumduol (PRD) and Thmar Krem (TMK) harvested during the wet season of 2013 at the Cambodian Agricultural Research and Development Institute. Orange graphs refer to saturated fatty acids whereas blue graphs refer to unsaturated fatty acids

The fatty acid profile of the mapping population falls in the range of C12-C24 (Fig. 4a). The major fatty acids in milled rice are the unsaturated fatty acids oleic acid (C18:1*n*-9) and linoleic acid (C18:2*n*-6) (Fig. 4a). Percentage compositions of fatty acids are as follows: C18:1*n*-9 (36.8%±1.4), C18:2*n*-6 (33.8%±1.2), palmitic acid (C16:0) (21.4%±0.8), stearic acid (C18:0) (3.2%±0.4), linolenic acid (C18:3*n*-3) (1.5%±0.1), and the rest were present at less than 1% in the samples (Fig. 4a). Significantly strong statistical correlations were observed between the most abundant fatty acid C18:1*n*-9 and the four other major fatty acids measured across 210 RILs in the mapping population (Fig. 4b). The strongest of these relationships was with C18:2*n*-6 (*r*=-0.74), followed by C16:0 (*r*=-0.53). No statistically significant relationship was observed between C18:1*n*-9 and the rest of the fatty acids and between C16:0 and C18:2*n*-6 in the mapping population (Fig. 4b). However, C16:0 was significantly negatively correlated with the % composition of C18:3*n*-3 (*r*=-0.41) (Fig. 4b). The frequency distributions of these fatty acids were normal (Fig. 5), with each parental line PRD and TMK falling within each trait distribution.

### Metabolite QTLs in the Recombinant Inbred Line Population

#### MQTLs for Volatile Compounds

**Fig. 6:**
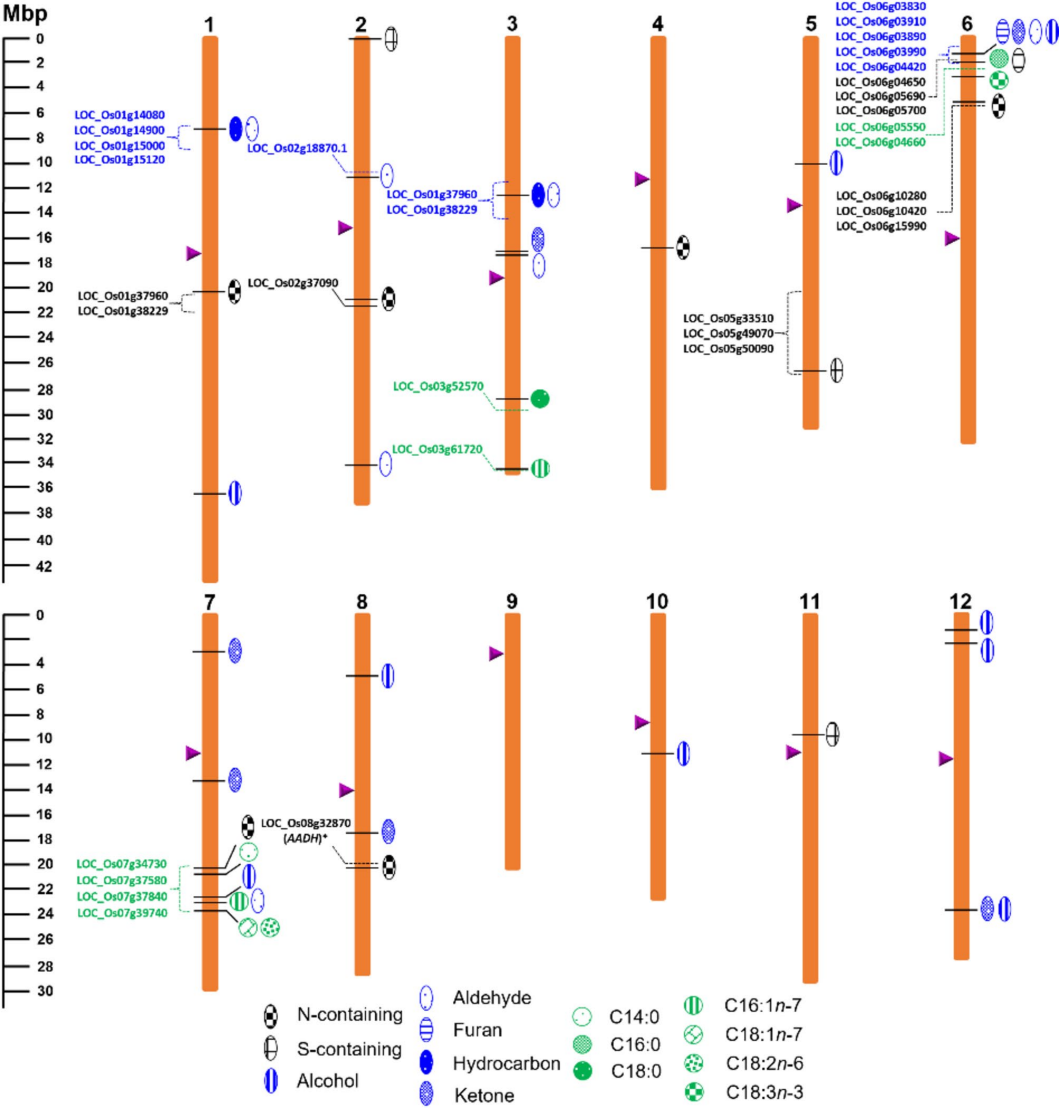
Physical map of metabolite QTLs (mQTL) identified for volatile compounds and fatty acids mapped on 12 rice chromosomes. These mQTLs were identified using GLM and CIM methods. On the left, the scale indicates the mega base pair (Mbp) distance. Arrows (in plum) on chromosomal maps represent the position of the centromere. Dashed lines and braces indicate the putative genes potentially associated with the mQTL

Using 768 SNP markers identified as polymorphic within the RIL population (Concepcion et al. 2019), several mQTLs with LOD ≥ 3 for odour-active volatile compounds were identified across the 12 rice chromosomes (Table 2). Thirty-three mQTLs passed the permutation tests at *P* = 0.05 as depicted in bold values. Sixteen volatile compounds associated with an mQTL were confirmed using analytical standards (Table 2; Table S1). The mQTL associated with the peak area of 2AP was identified on chromosome 8 (LOD = 50.28) at marker 8,834,914 (C/T) (position = 20,536,561 bp). Likewise, the same mQTL peak was identified for the other N-containing compounds — pyrrole, 6M5OTP and 2-acetylpyrrole. This common mQTL on chromosome 8 was statistically significant for all four compounds after the 1000 permutation iterations (*P* = 0.05). About 200 kbp away from the mQTL at chromosome 8, a protein-coding gene LOC_Os08g32870 (position = 20,379,794 to 20,386,061 bp), annotated as the *betaine aldehyde dehydrogenase 2* (*BADH2*) gene with transcript Os08t0424500-01 that codes for amino aldehyde dehydrogenase (AADH) was located, confirming the identity of the mQTLs (Table S3; Fig. 6). Candidate genes for the mQTLs on chromosomes 1, 2 and 6 include hydrolases and dehydrogenases, which are all expressed in rice endosperm and are involved in amino acid metabolism. Moreover, co-localised QTLs for these N-containing compounds were also identified on chromosomes 1, 2, 4, 6 and 7 (Table 2). Except for the mQTLs for 2-acetylpyrrole on chromosomes 1 and 6, all mQTLs identified for these N-containing compounds are significant after permutation analyses (Table 2). On the other hand, the mQTLs on chromosomes 4 and 7 are minor mQTLs (LOD ≥ 2.5) (Table 2) and were not statistically significant after permutation analyses. Several mQTLs were detected as common for volatile compounds derived from the oxidation of C18:1*n*-9 acid and C18:2*n*-6 (Table 3). Among these common mQTLs, the mQTL on chromosome 6 was associated with 13 volatile compounds encompassing aliphatic aldehydes, alcohols, and alkylfurans, which are mostly C18:2*n*-6 oxidation volatiles (Table 3). Meanwhile, the mQTL on chromosome 1 (position = 7.92 Mbp), was common for C18:1*n*-9 oxidation volatiles including nonanal, octanal, heptanal, nonane and 1-decene (SNP position = 7.92–11.16 Mbp) (Table 3). Genes shortlisted for this mQTL included LOC_Os01g14080 and LOC_Os01g15000, both annotated as lipases, LOC_Os01g14900 with a glycerol-3-phosphate acyltransferase activity, and LOC_Os01g15120, with hydrolase activity (Table S3). Nonanal, octanal, heptanal and nonane, were also associated with a common mQTL on chromosome 3 (marker = 277099; position = 12.01 Mbp) (Table 2); however, these mQTLs did not pass permutation analyses, hence were not further investigated for candidate genes.

**Table 2:** Quantitative trait loci detected for odour-active volatile compounds in 290 F_6_ rice progenies derived through a generalised linear model (GLM)

Trait	Chr	Marker Interval	QTL Peak				
			Marker	Position (Mbp)	LOD GLM	GLM Additive effect (DPE)	GLM PEV *R*^2^ (%)
**N-containing**							
2-Acetyl-1-pyrroline*	1	id1011000-id10113342	677,182	20.19	**8.45**	42776.11 (P)	14.9
	2	-	2,072,287	21.9	**19.12**	49942.26 (P)	27.48
	4	-	4,314,701	17.91	3.17	33670.39 (P)	5.69
	6	5,829,358–5,901,730	5,970,592	5.34	**11.82**	40754.65 (P)	19.74
	7	-	7,711,528	20.51	2.95	21798.14 (P)	4.63
	8	8,686,009-id8006926	8,834,914	20.54	**50.28**	74800.59 (P)	55.5
6-Methyl-5-oxo-2,3,4,5-tetrahydropyridine*	1	id1011000-id1013342	677,182	20.19	**6.19**	2513.63 (P)	10.9
	2	-	2,072,287	21.9	**13.9**	2985.45 (P)	20.8
	4	-	4,776,434	34.1	2.75	1432.68 (P)	4.35
	6	5,829,358–5,901,730	5,970,592	5.34	**9.23**	2297.54 (P)	15.5
	7	-	7,711,528	20.51	2.88	1459.74 (P)	5
	8	8,662,074-id8006926	8,834,914	20.54	**34.41**	4413.35 (P)	42.5
Pyrrole*	1	id1011000-id1013342	677,182	20.19	**7.6**	1358.27 (P)	13.4
	2	-	2,072,287	21.9	**18.1**	1733.23 (P)	26.23
	4	-	4,314,701	17.91	2.55	1005.60 (P)	4.43
	6	5,829,358–5,901,730	5,970,592	5.34	**9.54**	1376.86 (P)	16
	7	-	7,072,228	4.43	3.5	581.59 (P)	5.48
	8	8,662,074-id8006926	8,834,914	20.54	**51.66**	2592.63 (P)	56.5
2-Acetylpyrrole*	1	id1011000-id1013342	677,182	20.19	3.76	765.50 (P)	6.3
	2	-	2,072,287	21.9	**7.8**	889.64 (P)	12.3
	4	-	4,776,434	34.1	2.43	519.78 (P)	3.85
	6	5,828,525–6,030,751	5,970,592	5.34	4.68	467.54 (P)	7.7
	7	-	7,711,528	20.51	2.5	521.44 (P)	3.94
	8	8,662,074-id8006926	8,834,914	20.54	**14.75**	1183.59 (P)	21.1
**Alcohols**							
1-Butanol	5	-	5,121,882	8.84	3.76	74246.25 (T)	5.1
	7	-	7,782,655	22.45	3.06	43243.06 (P)	4.81
	8	8,149,888–8,201,100	8,149,888	5.28	3.24	55054.09 (T)	5.09
1-Pentanol*	6	5,828,525–5,901,730	SNP-6_1500959	1.5	**9.18**	26445.16 (P)	13.74
1-Hexanol	1	113,603–1,176,444	1,170,870	36.08	3.87	29316.23 (P)	6.05
	6	5,828,525–5,901,730	SNP-6_1500959	1.5	**11.06**	56060.90 (P)	16.31
	12	-	12,052,896	2	4.02	30351.20 (P)	6.63
1-Heptanol*	6	id600911-5926489	SNP-6_1500959	1.5	3.74	1405.10 (P)	5.84
	10	-	10,461,895	11.84	3.49	244.00 (P)	5.47
	12	-	12,072,314	2.65	4.34	1041.95 (P)	6.75
1-Octanol	6	5,830,047–5,926,489	SNP-6_1500959	1.5	**5.48**	2108.45 (P)	8.44
2*Z*-Octen-1-ol	12	-	id12008557	24.09	3.64	664.60 (T)	4.63
**Aldehydes**							
Pentanal*	6	5,828,528–5,901,730	SNP-6_1500959	1.5	3.89	28241.54 (P)	6.07
Hexanal*	6	5,828,528–5,901,730	SNP-6_1500959	1.5	**7.46**	388946.44 (P)	11.32
Heptanal*	1	-	245,712	7.92	**5.23**	7814.77 (P)	8.3
	3	-	2,770,990	12.01	3.08	8277.66 (P)	4.76
Octanal*	3	-	2,770,990	12.01	3.42	6152.02 (P)	5.35
	1	-	245,712	7.92	**5.1**	5815.93 (P)	8.6
Nonanal*	1	-	245,712	7.92	3.75	17858.83 (P)	6.1
	3	-	2,770,990	12.01	3.19	21380.35 (P)	4.95
2*E*-Nonenal	6	5,828,525–6,030,751	id6001535	2.01	**9.94**	2525.56 (P)	14.8
2*E*-Octenal	6	5,828,525–6,030,751	SNP-6_1500959	1.5	**8.01**	1335.54 (P)	12.1
Dodecanal	2	-	1,711,443	11.05	**5.49**	82.81 (T)	8.46
2-Methyl propanal	6	SNP-6_1500959–5,901,730	5,883,472	2.13	3.26	94300.74 (T)	5.12
3-Methyl butanal	7	-	rd7002211	23.31	3.03	54189.23 (T)	4.92
2-Ethyl hexanal	2	2,350,752–2,449,332	ud2002015	34.04	3.95	2148.11 (P)	6.16
2*E*,4*E*-Nonadienal	6	5,849,924–5,882,239	SNP-6_1500959	1.5	**6.48**	660.81 (P)	9.91
Benzaldehyde	3	2,902,136–2,997,078	2,945,947	17	3.1	6844.31 (P)	4.87
**Ketones**							
2-Heptanone*	6	5,828,525–5,901,730	SNP-6_1500959	1.5	**10.42**	25213.90 (P)	15.45
2-Decanone	3	-	2,770,990	12.01	3.26	659.12 (P)	5.07
	4	-	4,754,818	33.16	3.75	320.71 (T)	5.86
	6	-	6,794,969	25.76	3.34	624.49 (P)	5.01
	7	-	7,040,326	3.11	3.95	798.38 (P)	6.48
3-Heptanone	7	-	7,457,807	13.82	4.09	1987.37 (P)	6.48
3-Octanone	7	-	rd7002211	23.31	3.74	137.41 (P)	6.03
2,3-Butanedione*	3	SNP-23_0257466–3,171,256	3,096,758	20.77	3.6	48429.60 (P)	4.57
	8	-	8,686,009	17.32	3.04	9226.31 (T)	4.78
3-Octen-2-one	6	-	SNP-6_1500959	1.5	**6.78**	1435.75 P)	10.34
3-Penten-2-one	12	1,298,505–13,010,827	id12008557	24.09	3.95	8371.62 (T)	5.08
**Furans**							
2-*n*-Butylfuran	6	5,828,525–5,901,730	SNP-6_1500959	1.5	**10.65**	2989.19 (P)	15.75
2-Pentylfuran*	6	5,828,525–5,926,489	SNP-6_1500959	1.5	**10.02**	18417.44 (P)	14.9
**S-containing**							
Dimethyl disulfide*	2	id2000235-id2001102	id2000235	0.32	3	13657.27 (T)	4.72
	6	id6000606-5901730	5,883,472	2.13	**8.04**	23115.01 (T)	12.14
Dimethyl trisulfide*	5	id5012179-id5014124	5,754,154	27.84	**5.4**	1985.81 (T)	8.34
	6	SNP-6_1500959–5,901,730	5,882,239	2.01	**6.46**	2260.91 (T)	9.88
	11	-	c11p10263123	10.26	3.11	1378.45 (T)	4.53
**Hydrocarbons**							
Nonane	1	-	245,712	7.92	**12.88**	31443.46 (P)	21.8
	3	id3004190-2838381	2,770,990	12.01	3.5	23273.62 (T)	5.49
1-Decene	1	-	245,712	7.92	**6.55**	33866.11 (P)	8.8

*Identity confirmed using analytical standards; Chr, chromosome number; Position (Mbp), marker physical position in mega base pairs; LOD GLM, Logarithm of Odds using generalised linear model; PEV (*R*^*2*^), Percentage of total phenotypic variance explained by the QTL; DPE, direction of phenotypic effect; P, Phka Rumduol; T, Thmar Krem; bold values for LOD GLM means significant at *P* = 0.05 after 1000 permutations. The marker interval not indicated here implies the significant association of the trait with a single marker

Sulfur-containing volatile compounds DMDS and DMTS were associated with a common mQTL on chromosome 6 (marker 5883472, position = 2.03 Mbp) (Table 2) that shows statistically significant LOD scores for DMDS (6.46) and DMTS (8.01) and explains 12.1% and 9.9% of the variation in the RIL population, respectively. The additive effect of this mQTL was contributed by TMK alleles (Table 2). DMTS was also associated with an mQTL on chromosome 5 (Table 2). Putative genes for DMDS and DMTS on chromosome 6 (Table S3) include the following: (1) LOC_Os06g04650 codes for peptide methionine sulfoxide reductase; (2) LOC_Os06g05690 that codes for the putative chloroplast/chromoplast precursor cysteine synthase (Os06t0149700); (3) LOC_Os06g05700 (position = 2,579,088 − 2,581,726 bp); and (4) LOC_Os06g07860 that codes for cystathionine gamma-synthase (Os06t0175800). Candidate genes potentially associated with DMTS accumulation on chromosome 5 include LOC_Os05g49070 and LOC_Os05g50090 coding for a putative and expressed dehydrogenase, and oxidoreductase, 2OG-FeII oxygenase domain containing, respectively. About seven Mbp away from the mQTL peak lies a gene that encodes for peptide methionine sulfoxide reductase, which was also shortlisted as a candidate gene for the mQTL for DMTS and DMDS on chromosome 6 (Table S3).

#### QTLs for Fatty Acid Percentage Composition

The composition of these fatty acids exhibits a normal distribution within the RIL population indicating the interplay of multiple genes (Fig. 5). Eight QTLs were identified for the fatty acids measured in the population (Table 3). From these QTLs, three were identified as common for % fatty acid composition (Table 3). The most significant among the QTLs for fatty acids are located on chromosomes 3, 6 and 7 (Table 3). A major mQTL was identified for palmitic acid (C16:0) located on chromosome 6 and peaked at SNP marker 5,882,239 (position = 2.03 Mbp) and explains 41.1% of the fatty acid variation in the population. Near this genetic region is a QTL for linolenic acid (C18:3*n*-3), which is additively contributed by the TMK allele (Table 3). Genes shortlisted for C16:0 and C18:3*n*-3 mQTLs on chromosome 6 are GDSL-like lipase/acylhydrolase (LOC_Os06g05550; position = 2,515,760–2,517,940 bp) and a putative oxidoreductase, 2OG-Fe oxygenase family protein (LOC_Os06g04660; position = 2,030,467-2,034,813 bp) (Fig. 6; Table S3). QTLs common to the most predominant fatty acids — oleic acid (C18:1*n*-9) and linoleic acid (C18:2*n*-6) — were identified on chromosome 7 (position = 23,785,728 bp; marker = 7820873) (Table 3). This QTL explains 32% of the variation in % fatty acid composition. Increased C18:1*n*-9 is contributed by PRD, whereas increased C18:2*n*-6 is contributed by TMK (Table 3).

**Table 3:** Quantitative trait loci detected for % fatty acid composition in 210 F_6_ rice progenies derived through generalised linear model (GLM)

*Trait	Chr	Marker interval	QTL Peak					
			Marker	SNP	Position (Mbp)	LOD GLM	GLM Additive effect (DPE)	GLM PEV *R*^2^ (%)
C16:1*n*-7*	3	3,538,410 − 3,495,083	3,528,886	G/A	34.92	**4.57** ^**b**^	0.01 (P)	10.3
C18:0*	3	3,327,551–3,430,019	id3013770	A/G	29.26	**5.17** ^**b**^	0.12 (P)	11.5
C16:0*	6	5,828,525–5,926,489	5,882,239	A/G	2.03	**22.28** ^**a**^	0.54 (P)	41.1
C18:3*n*-3*	6	5,849,924–5,926,489	5,926,489	T/C	3.72	**4.70** ^**b**^	0.05 (T)	10.6
C14:0*	7	7,619,926–7,820,873	7,746,043	C/T	21.34	**4.17** ^**c**^	0.02 (P)	9.4
C16:1*n*-7*	7	7,769,958–7,898,771	7,809,062	G/A	23.36	**6.84** ^**a**^	0.01 (T)	15
C18:1*n*-9	7	7,669,390–7,898,771	7,820,873	G/T	23.79	**7.89** ^**a**^	0.60 (P)	17.1
C18:2*n*-6*	7	7,769,958–7,898,771	7,820,873	G/T	23.79	**6.73** ^**a**^	0.49 (T)	14.8

*Identity confirmed using analytical standards; Chr, chromosome number; Position (Mbp), marker physical position in mega base pairs; LOD GLM, Logarithm of Odds using generalised linear model; PEV (*R*^*2*^), Percentage of total phenotypic variance explained by the QTL; DPE, direction of phenotypic effect; P, Phka Rumduol; T, Thmar Krem. ^a, b,c^QTLs identified using GLM above the *p* = 0.001, *p* = 0.01 and *p* = 0.05 threshold using permutation analysis, respectively

## Discussion

Of the 131 compounds, 88 were identified or putatively identified in rice and have known odour threshold values and descriptions that are relevant to the sensory and eating attributes of different rice varieties and food products (Table 1) (Buttery et al. 1983, 1988, 1999; Calingacion et al. 2017; Concepcion et al. 2018; Daygon et al. 2016; Mumm et al. 2016). Dodecanal and 1-decene have not been reported in rice but were included in the list (Table 1) since these are odour-producing compounds (Ahmed et al. 1978; D’auria et al. 2022; Guadagni et al. 1963).

### Confirmation of mQTLs for 2AP and Derivatives

The presence and variable peak abundance of N-containing compounds are strongly controlled by a major gene on chromosome 8, which verifies previous findings on the genetics of 2AP (Kovach et al. 2009) and its derivatives (Daygon et al. 2017). The strong correlations among these N-containing compounds and their common genetic link validated their common biochemical link (Daygon et al. 2017) in a RIL *indica* rice population developed from varieties with contrasting fragrance-associated traits, which has not been done before. In addition to the mQTL associated with *AADH* at chromosome 8, and the presence of several mutations in this gene (Kovach et al. 2009; Shao et al. 2011; Shi et al. 2008), previous QTL mapping studies have also identified QTLs for aroma at other chromosomes, such as on chromosome 3 (Singh et al. 2007), 4 (Golestan Hashemi et al. 2015), 3 and 4 (Amarawathi et al. 2008); and 4 and 12 (Lorieux et al. 1996) and more recently on chromosome 1 (Daygon et al. 2017). These past studies, and the novel mQTLs for 2AP, pyrrole, 2-acetylpyrrole and 6M5OTP at chromosomes 2 and 6 identified here, indicate the vast diversity in the genetics underpinning 2AP and suggest that further investigation is needed to fully understand the underlying genetic basis of these fragrance compounds. Different combinations of alleles at these loci could contribute to our understanding of how some rice varieties are consistently low on 2AP, some are medium and a few are high like PRD. These identified mQTLs and the minor mQTLs co-localised on chromosomes 4 and 7 can be further validated by fine mapping.

### Markers for Cooked Rice Off-Flavours

Markers to select against undesirable quality traits are complementary to markers for desirable traits to develop a rice variety that maintains its desirable fragrance and resists oxidation for longer periods. Volatile S-containing compounds such as the simplest disulfides DMTS and DMDS originate from S-containing amino acids or proteins (Parker 2015). These compounds are produced from the thermal degradation of the naturally occurring S-containing amino acid, S-methyl-L-cysteine, and its sulfoxide. DMDS is the predominant volatile compound generated from both precursors, whereas DMTS is an odour-active breakdown product of S-L-methyl cysteine sulfoxide (Kubec et al. 1998; Parker 2015). These compounds exhibit strong aromas and have been identified and studied in rice and rice-derived foods (Buttery et al. 1999; Daygon et al. 2016) and other plant products (Elmore et al. 2008; Gonda et al. 2013). DMTS, known to have an alliaceous type or “garlic-like” odour (Table 1), has been reported to be a major cause of undesirable flavour in overcooked vegetables and other plant foods (McGorrin 2011). The identity of DMTS and DMDS was confirmed using analytical standards (Table S1). Identification of mQTLs for these two compounds suggests these traits vary across rice genotypes (Table 3; Fig. 6). Given these S-containing compounds are found in both fragrant and non-fragrant rice, there is an indication all rice genotypes produce these undesirable odours; however, the potency of these odours depends on their concentration and odour threshold values relative to the concentration of compounds with desirable aromas that possibly mask the undesirable aromas or vice versa (McGorrin 2011).

Another group of putatively identified amino acid-derived volatile compounds with undesirable odours includes four aldehydes — 2-ethyl hexanal, 2-methyl butanal, 2-methyl propanal and 3-methyl butanal. 2-Ethyl hexanal is associated with a sharp and powerful odour and was previously identified in rice (Calingacion et al. 2015), although its identity was not confirmed using an analytical standard. 2-Methyl propanal, 2-methyl butanal, and 3-methyl butanal are known as Strecker aldehydes and are formed from the amino acids valine, isoleucine, and leucine, respectively (Belitz et al. 2004; Rizzi 1999). These three volatile aldehydes have been previously identified as significant compounds that differentiate wheat cultivars with different sulfur nutrition (Elmore et al. 2008). 2-Methyl butanal and 2-methyl propanal are associated with distinct malty flavours (Belitz et al. 2004; Moore et al. 1976) that can greatly contribute to the complexity of fragrance in rice.

### Evolution of Volatile Compounds from Lipids

The wealth of odour-active volatile compounds identified and putatively identified in this study shows the complexity of metabolites associated with signature aroma for a particular variety (Mumm et al. 2016). A wide array of volatile compounds is derived from the oxidation of lipids. With a small number of lipids present in milled rice (Resurreccion and Juliano 1975), the influence of lipids on rice quality can easily be ignored until the biochemical changes that explain the important quality traits such as texture (Concepcion et al. 2020) and aroma (Concepcion et al. 2018; Daygon et al. 2016) are carefully investigated. Oxidation of oleic and linoleic acids produces odour-active volatiles such as aldehydes, alcohols, and hydrocarbons, for example, as breakdown products of their monohydroperoxides (Christie 1973; Frankel 1983, 2012; Velasco et al. 2010). In addition, the evolution of these volatile compounds can be regulated by the action of lipoxygenase (LOX) and hydrolase enzymes such as lipases (Schwab et al. 2008). In rice seeds, LOX activity is localised in the bran and LOX-3 is the major isozyme component (Suzuki et al. 1999). Hexanal, a major marker for lipid oxidation (Schaich et al. 2013) exhibited strong correlations with other volatile compounds that are known lipid oxidation products (Frankel 1983), suggesting a biochemical link between these volatile compounds and their common fatty acid origin. This biochemical link has been described in a previous QTL mapping study using a RIL population derived from two non-fragrant rice varieties, where strong positive correlations were identified for hexanal, 1-pentanol and 2-heptanone (Calingacion et al. 2017); however, the mQTL was in chromosome 3 rather than chromosome 6.

Common mQTLs for unsaturated fatty acid oxidation volatile compounds (Table 3) suggest a common genetic and/or biochemical origin. A candidate gene on chromosome 6 that could explain the variation in these volatile compounds is a putative lipoxygenase (LOC_Os06g04420) that is homologous to LOX5 in *A. thaliana* (Umate 2011). However, no existing gene expression data for this gene is currently available. Lipoxygenase regions in chromosome 3 such as LOC_Os03g49380 (position = 28,106,903 − 28,113,300 bp), LOC_Os03g52860 (position = 30,315,455 to 30,318,972 bp) were too distant from the QTL identified in this study.

Oleic acid oxidation alcohols 1-heptanol and 1-hexanol were associated with a common QTL on chromosome 12, where LOC_Os12g05630 (position = 2,588,413–2,589,305 bp), a hypothetical protein (putative hydrolase) is in close proximity. Hydrolases are enzymes that catalyse the cleavage of a covalent bond using water (Sullivan 2004). This enzyme group includes esterases, such as phosphatases and lipases, that act on ester bonds, and proteases or peptidases that act on amide bonds in peptides. Alkylfurans are one of the most significantly discriminating classes of compounds in fragrant rice including PRD (Concepcion et al. 2018), therefore their presence and relative abundance were monitored in the RIL population. Furans are formed from the degradation and rearrangement of carbohydrates and polyunsaturated fatty acids upon high-temperature treatment (Crews and Castle 2007; Seok et al. 2015). The formation of furans from unsaturated fatty acids increases with increasing degree of unsaturation (Fan 2015; Perez and Juliano 1978). 2-Pentylfuran has been described to have a characteristic beany odour, whereas 2-ethylfuran and 2-*n*-butylfuran are described as having burnt and fruity aromas respectively (http://www.thegoodscentscompany.com). 2-Pentylfuran and 2-*n*-butylfuran are derived from unsaturated fatty acid oxidation, specifically from linoleic and linolenic acid, respectively. These two compounds are also associated with the same QTL on chromosome 6, suggesting a common biochemical mechanism for the evolution of these compounds. Coinciding with previous findings, 2-pentylfuran is highly correlated with hexanal, indicating a strong biochemical link between the two compounds (Griglione et al. 2015). Hexanal is also strongly correlated with 1-hexanol, which can be explained by the biochemical origin of 1-hexanol from hexanal (Matoba et al. 1989). Identifying the origin of these fatty acids, and specifically which lipid molecule, will provide insights into the underlying genetic mechanisms that govern the accumulation of these lipid species (Concepcion et al. 2020). This will also provide an understanding of the odour threshold values of these compounds and the extent to which they contribute to the aroma of fragrant rice PRD.

### Fatty Acids

The fatty acids identified in this current study coincide with previous studies on FAME analysis in rice grains (Goffman et al. 2003; Resurreccion and Juliano 1975). The predominance of unsaturated fatty acids oleic and linoleic acid in milled rice of the RIL population (Fig. 4a) indicates that the lipid origin of these fatty acids is mostly composed of oleic and linoleic acid as side chains. The main pathways associated with lipid biosynthesis in a rice grain involve the formation of free fatty acids, glycerolipids, glycerophospholipids, glycolipids and lysophospholipids (Christie 1973). Previous lipidomics studies on the RIL population used in this current study detected lipid species largely composed of glycerolipids and glycerophospholipids (Concepcion et al. 2020). Ying et al. (2012) identified several QTLs for fatty acid composition in brown rice using a double haploid population. Although this current research does not confirm previously reported QTLs for fatty acid composition, a similar trend in statistical correlations between the compositions of the major fatty acids in rice was observed consistent with previous findings of % fatty composition in different cereals including rice (Fig. 4b). Variation in unsaturated fatty acid % composition across the RIL population can be attributed to the inherent difference in overall composition of lipid species in grains of PRD and TMK. Genes shortlisted for variation in oleic and linoleic acid on chromosome 7 include the following: (i) acyltransferase, LOC_Os07g34730 (position = 20,812,544–20,808,033 bp), which is orthologous to genes for glycerolipid biosynthesis in Arabidopsis (AT5G60620), maize (GRMZM2G123987) and sorghum (Sb02g035260); (ii) putative diacylglycerol kinase, a critical signalling enzyme that phosphorylates diacylglycerol to yield phosphatidic acid (Escobar-Sepúlveda et al. 2017), which is a precursor to all glycerophospholipids as well as triacylglycerols and galactolipids (Arisz et al. 2013); (iii) putative lipase (LOC_Os07g37840); (iv) GDSL-like lipase/acylhydrolase (LOC_Os07g39740), and LOC_Os07g23410, which encodes a putative omega-6 fatty acid desaturase, located farther away from the peak of the mQTL (pos = 13,201,060 − 13,206,929) (Fig. 6; Table S3). Putative lipases (triacylglycerol ester hydrolases, EC 3.1.1.3) were likely involved in this variation of fatty acid composition (Table S3). Lipases are enzymes that catalyse the cleavage of ester bonds in triglycerides to yield glycerol and free fatty acids and are distributed broadly throughout plants, animals, and microorganisms (Santos et al. 2013). GDSL-like lipase/acylhydrolase and oxidoreductases exhibit hydrolysing functions that can be linked to the release of free fatty acids from an intact lipid molecule. GDSL-like lipases have been reported to play a role in plant systemic immunity (Hong et al. 2008), whereas oxidoreductase and 2OG-Fe oxygenase catalyse the formation of plant hormones and pigments such as flavones (Jiang et al. 2016). The presence of genes involved in lipid biosynthetic pathways close to the identified mQTLs for fatty acid % composition indicates a genetic origin for the variation in fatty acid composition in rice. Moreover, the identification of the actual lipid species will provide information on the origin of these fatty acids.

## Conclusions

This research study identified several mQTLs for odour-active volatile compounds with desirable and off-flavoured aromas in a rice mapping population derived from two rice varieties with different compositions of volatile metabolites and fatty acids. These mQTLs can be used as selection markers to identify breeding lines and new varieties with the quality profile of PRD and with the added tolerance to abiotic stress and resistance to pests. This research has demonstrated that breeding for high-quality rice can be further improved with the current tools in separation science and molecular biology. The data and knowledge obtained from this research will be valuable to both rice breeders and rice quality researchers.

## Electronic Supplementary Material

Below is the link to the electronic supplementary material.

Supplementary Material 1

## Data Availability

The datasets used and/or analysed during the current study are available from the corresponding author upon reasonable request.
