# Supplementary material for: Metabolite Quantitative Trait Loci Mapping for Fragrance and Fatty Acid Composition in Rice (Oryza Sativa L.)
Source: Rice (N Y). 2025 Oct 22;18:96. doi: 10.1186/s12284-025-00790-8 (PMC12546234; doi:10.1186/s12284-025-00790-8)
Supplement: Supplementary file 1 — Supplementary Material 1 [file 12284_2025_790_MOESM1_ESM.docx]

Rice (Concepcion et al.)

Metabolite quantitative trait loci mapping for fragrance and fatty acid composition in rice (*Oryza sativa* L.)

Supporting information

**Author names and affiliations:**

Jeanaflor Crystal T. Concepcion^1,3,*^, Mary J. Garson^2^, and Melissa A. Fitzgerald^1,4^

JCT Concepcion (ORCID: 0000-0003-4524-2794)

MJ Garson (ORCID: 0000-0001-8670-1075)

MA Fitzgerald (ORCID: 0000-0002-0761-764X)

^1^ School of Agriculture and Food Sustainability, The University of Queensland, Brisbane, Australia 4072

^2^ School of Chemistry and Molecular Biosciences, The University of Queensland, Brisbane, Australia 4072

^3^ Current Address: Carle R. Woese Institute for Genomic Biology, University of Illinois at Urbana-Champaign, Urbana, Illinois, United States 61801

^4^ Current Address: University of Al Dhaid, Al Dhaid, Sharjah, United Arab Emirates

* Correspondence: [jcc1@illinois.edu](mailto:jcc1@illinois.edu)

**Table S1.** List of analytical standards in the untargeted profiling of volatile compounds in rice using two-dimensional gas chromatography time-of-flight mass spectrometry.

| **Standard** | **PubChem CID** | **Manufacturer** | **Purity (%)** | **Retention time (primary column, secondary column) (s)** |
| --- | --- | --- | --- | --- |
| Aldehydes | | | | |
| Butanal | 261 | Sigma-Aldrich | 99 | 267.5 , 0.640 |
| Furfural | 7362 | Sigma-Aldrich | 99 | 582.5 , 1.710 |
| Heptanal | 8130 | Sapphire Biosciences | 95 | 615.0 , 1.710 |
| Hexanal | 6184 | Sigma-Aldrich | 98 | 497.5 , 0.695 |
| Nonanal | 31289 | Sigma-Aldrich | 98.5 | 830.0 , 0.795 |
| Octanal | 454 | Sigma-Aldrich | 99 | 725.0 , 0.735 |
| Pentanal | 8063 | Sigma-Aldrich | 97 | 377.5 , 0.690 |
| 2,4-Heptadienal | 5283321 | Sigma-Aldrich | 90 | 730.0 , 0.585 |
| 2-Pentenal | 5364752 | Sigma-Aldrich | ≥ 95.0 | 450.0 , 0.500 |
| 2-Heptenal | 5283316 | Sigma-Aldrich | > 95.0 | 677.5 , 0.775 |
| 2-Octenal | 5283324 | Sigma-Aldrich | ≥ 97.0 | 767.5 , 0.520 |
| 4-Heptenal | 5362814 | Sigma-Aldrich | ≥ 98.0 | 600.0 , 0.480 |
| Ketones | | | | |
| 2,3-Butanedione | 650 | Sigma-Aldrich | 97 | 275.0 , 1.065 |
| 2-Heptanone | 8051 | Sigma-Aldrich | 99 | 607.5 , 0.710 |
| 2-Octanone | 8093 | Sigma-Aldrich | 98 | 715.0 , 0.710 |
| 2-Pentanone | 7895 | Sigma-Aldrich | 98–100 | 355.0 , 0.420 |
| 1-Octen-3-one | 61346 | Sigma-Aldrich | ≥ 97.0 | 682.5 , 0.490 |
| 2-Undecanone | 8163 | Sigma-Aldrich | 99 | 1005.5 , 1.075 |
| 4-Methyl-2-pentanone | 7909 | Sigma-Aldrich | 98.5 | 582.5 , 0.950 |
| Alcohols | | | | |
| 1-Butanol | 19422 | Sigma-Aldrich | ≥ 99.9 | 355.0 , 0.555 |
| 1-Pentanol | 6276 | Sigma-Aldrich | 99 | 470.0 , 0.930 |
| 1-Heptanol | 8129 | Sigma-Aldrich | > 99 | 695.0 , 0.865 |
| 2-Methyl-1-pentanol | 7745 | Sigma-Aldrich | 99 | 555.0 , 1.180 |
| 2-Octanol | 20083 | British Drug House | 97 | 737.5 , 0.785 |
| 2-Penten-1-ol | 5364919 | Sigma-Aldrich | ≥ 96 | 482.5 , 0.635 |
| 1-Octen-3-ol | 18827 | Sigma-Aldrich | ≥ 98.0 | 690.0 , 0.535 |
| 4-Ethylcyclohexanol | 11524 | Sigma-Aldrich | 98 | 790.0 , 0.570 |
| Hydrocarbons | | | | |
| Dodecane | 8182 | Supelco | 99.8 | 877.5 , 0.645 |
| Heptane | 8900 | Sigma-Aldrich | 99 | 345.0 , 0.575 |
| Hexane | 8058 | Sigma-Aldrich | 95 | 225.0 , 0.645 |
| Limonene | 440917 | Sigma-Aldrich | 98 | 732.5 , 0.655 |
| Nonane | 8141 | Sigma-Aldrich | 98 | 570.0 , 0.550 |
| Octane | 356 | Sigma-Aldrich | 98 | 460.0 , 0.550 |
| Tetradecane | 12389 | Supelco | 99.8 | 1035.0 , 0.645 |
| Tridecane | 12388 | Sigma-Aldrich | 99 | 975.0 , 0.790 |
| Undecane | 14257 | Sigma-Aldrich | 99 | 782.5 , 0.585 |
| 3-Octene | 638228 | Sigma-Aldrich | 98 | 445.0 , 0.355 |
| Aromatic hydrocarbons | | | | |
| p-Xylene | 7809 | Sigma-Aldrich | ≥ 99.5 | 570.0 , 0.455 |
| Ethylbenzene | 7500 | Sigma-Aldrich | ≥ 99.5 | 545.0 , 0.440 |
| Furan derivatives | | | | |
| 2-Pentylfuran | 19602 | Sigma-Aldrich | > 98 | 690.5 , 0.690 |
| 2-Methylfuran | 534225 | Sigma-Aldrich | > 99 | 262.5 , 0.800 |
| S-containing | | | | |
| Dimethyl trisulfide | 19310 | Sigma-Aldrich | ≥ 98.5 | 707.5 , 1.060 |
| N-containing | | | | |
| 2-Acetyl-1-pyrroline | 522834 | ChemStep | 95 | 647.5 , 1.040 |
| *6M5OTP |  | Sigma-Aldrich |  | 705.0 , 1.140 |
| Pyrrole | 8027 | Fluka | > 99 | 532.5 , 1.480 |
| Pyridine | 1049 | Sigma-Aldrich | 99.9 | 437.5 , 0.915 |

*Synthesised at the School of Chemistry and Molecular Biosciences, The University of Queensland.

**Table S2.** Volatile compounds detected in the headspace of milled rice lines derived from the cross between PRD and TMK, their molecular formula, molecular weight, unique mass and % similarity in mass spectral fragmentation compared to the standards in-house* and the NIST library†.

| **Name** | **Molecular Formula** | **Molecular weight (g mol^-1^)** | **Unique Mass** | **Similarity (%)** | **R.T. (s)**  **(1^st^, 2^nd^)** |
| --- | --- | --- | --- | --- | --- |
| 1-Butanol † | C4H10O | 74.12 | 56 | 93.3 | 357.5 , 1.135 |
| 3-Methyl-1-butanol † | C5H12O | 88.15 | 55 | 90.4 | 440.0 , 1.095 |
| 1-Decene† | C10H20 | 140.27 | 55 | 81.5 | 680.0 , 0.690 |
| 1-Heptanol * | C7H16O | 116.20 | 70 | 92.2 | 695.0 , 0.865 |
| 1-Hexanol † | C6H14O | 102.17 | 56 | 94.5 | 592.5 , 1.070 |
| 2-Ethyl-1-hexanol † | C8H18O | 130.23 | 57 | 95.1 | 765.0 , 0.980 |
| 1-Octanol † | C8H18O | 130.23 | 56 | 93.9 | 805.0 , 1.005 |
| 2-Butyl-1-octanol † | C12H26O | 186.33 | 57 | 85.7 | 1100.0 , 0.700 |
| 1-Octen-3-ol * | C8H16O | 128.21 | 57 | 87.8 | 690.0 , 0.535 |
| 3,7-Dimethyl-1-octene † | C10H20 | 140.27 | 71 | 84.7 | 775.0 , 0.670 |
| 1-Pentanol * | C5H12O | 88.15 | 55 | 94.1 | 470.0 , 0.930 |
| 1-Penten-3-ol † | C5H10O | 86.13 | 57 | 81.9 | 377.5 , 1.120 |
| 1-Propanol † | C3H8O | 60.10 | 59 | 93.4 | 250.0 , 1.110 |
| 2-Methyl-1-propanol † | C4H10O | 74.12 | 41 | 86.4 | 320.0 , 1.085 |
| 2,3-Butanedione * | C4H6O2 | 86.09 | 86 | 96.1 | 275.0 , 1.065 |
| 2*E*,4*E*-Nonadienal † | C9H14O | 138.21 | 81 | 80.0 | 965.0 , 1.015 |
| 2-Acetylpyrrole* | C6H7NO | 109.13 | 94 | 91.9 | 842.5 , 2.370 |
| 2-Acetyl-1-pyrroline* | C6H9NO | 111.14 | 83 | 88.0 | 647.5 , 1.040 |
| 2-Butanol † | C4H10O | 74.12 | 45 | 82.7 | 287.5 , 0.990 |
| 2-Butanone † | C4H8O | 72.11 | 43 | 93.8 | 277.5 , 0.825 |
| 2-Decanone † | C10H20O | 156.26 | 58 | 94.3 | 922.5 , 0.840 |
| 2-Heptanone* | C7H14O | 114.19 | 58 | 95.6 | 607.5 , 0.710 |
| 2*Z*-Heptenal † | C7H12O | 112.17 | 83 | 89.5 | 700.0 , 0.930 |
| 2-Hexanol † | C6H14O | 102.17 | 45 | 91.0 | 510.0 , 0.975 |
| 2-Hexanone † | C6H12O | 100.16 | 43 | 90.5 | 497.5 , 0.845 |
| 2-Hexenal † | C6H10O | 98.14 | 83 | 95.5 | 582.5 , 0.94 |
| 2-*n*-Butylfuran † | C8H12O | 124.18 | 81 | 85.4 | 587.5 , 0.815 |
| 2-Nonanone † | C9H18O | 142.24 | 55 | 88.9 | 820.0 , 0.830 |
| 2*E*-Nonenal † | C9H16O | 140.22 | 56 | 89.9 | 902.5 , 0.990 |
| 2-Octanone* | C8H16O | 128.21 | 58 | 91.9 | 715.0 , 0.710 |
| 2*E*-Octen-1-ol † | C8H16O | 128.21 | 57 | 83.7 | 900.0 , 0.830 |
| 2*E*-Octenal † | C8H14O | 126.20 | 70 | 91.6 | 805.0 , 0.920 |
| 2-Pentanol † | C5H12O | 88.15 | 45 | 89.0 | 392.5 , 1.000 |
| 2-Pentanone † | C5H10O | 86.13 | 43 | 92.5 | 375.0 , 0.840 |
| 4-Hydroxy-4-methyl-2-pentanone † | C6H12O2 | 116.16 | 59 | 80.0 | 590.0 , 1.180 |
| 2-Methyl-2-propanol † | C4H10O | 74.12 | 59 | 86.0 | 217.5 , 0.855 |
| 2-Undecanone † | C11H22O | 170.29 | 58 | 94.0 | 1110.0 , 1.115 |
| 3*Z*-Dodecene † | C12H24 | 168.32 | 83 | 83.8 | 1137.5 , 0.720 |
| 3-Octanone † | C8H16O | 128.21 | 56 | 80.0 | 712.5 , 0.830 |
| 3*E*-Octen-2-one † | C8H14O | 126.20 | 55 | 85.6 | 785.0 , 0.910 |
| 3-Penten-2-one † | C5H8O | 84.12 | 69 | 81.4 | 442.5 , 0.780 |
| 3*Z*-Tridecene † | C13H26 | 182.35 | 41 | 81.3 | 992.5 , 0.730 |
| 6M5OTP* | C5H9N | 83.13 | 55 | 82.8 | 705.0 , 1.140 |

**Table S2.** Continued..

| **Name** | **Molecular Formula** | **Molecular weight (g mol^-1^)** | **Unique Mass** | **Similarity (%)** | **R.T. (s)**  **(1^st^, 2^nd^)** |
| --- | --- | --- | --- | --- | --- |
| Acetoin † | C4H8O2 | 88.11 | 45 | 86.3 | 432.5 , 1.460 |
| Acetophenone † | C8H8O | 120.15 | 105 | 90.6 | 830.0 , 1.245 |
| α-Methylstyrene † | C9H10 | 118.18 | 118 | 90.8 | 702.5 , 0.935 |
| α-Phellandrene † | C10H16 | 136.23 | 93 | 92.3 | 715.0 , 0.770 |
| α-Pinene † | C10H16 | 136.23 | 93 | 92.8 | 635.0 , 0.715 |
| Aromandendrene † | C15H24 | 204.35 | 73 | 87.2 | 1122.5 , 0.815 |
| Benzaldehyde † | C7H6O | 106.12 | 106 | 94.9 | 715.0 , 1.280 |
| Benzene † | C6H6 | 78.11 | 78 | 94.5 | 330.0 , 0.835 |
| 1,2,3-Trimethyl benzene † | C9H12 | 120.19 | 105 | 94.0 | 715.0 , 0.875 |
| Propyl benzene † | C9H12 | 120.19 | 91 | 89.3 | 667.5 , 0.845 |
| Benzophenone † | C13H10O | 182.22 | 105 | 90.8 | 1085.0 , 0.315 |
| Benzothiazole † | C7H5NS | 135.19 | 135 | 90.5 | 990.0 , 1.485 |
| Butanal * | C4H8O | 72.11 | 72 | 87.6 | 267.5 , 0.640 |
| 2-Methyl butanal † | C5H10O | 86.13 | 57 | 84.0 | 345.0 , 0.790 |
| 3-Methyl butanal † | C5H10O | 86.13 | 41 | 90.5 | 335.0 , 0.800 |
| Camphene † | C10H16 | 136.23 | 93 | 93.7 | 657.5 , 0.735 |
| Cyclopentanone † | C5H8O | 84.12 | 55 | 80.0 | 515.0 , 1.020 |
| Decanal † | C10H20O | 156.26 | 57 | 91.3 | 932.5 , 0.845 |
| Dimethyl trisulfide * | C2H6S3 | 126.30 | 126 | 90.5 | 707.5 , 1.060 |
| Dimethyl disulfide † | C2H6S2 | 94.20 | 94 | 96.5 | 435.0 , 0.930 |
| Dodecanal † | C12H24O | 184.32 | 57 | 91.2 | 1107.5 , 0.685 |
| Dodecane * | C12H26 | 170.33 | 57 | 95.1 | 877.5 , 0.685 |
| Ethylbenzene † | C8H10 | 106.16 | 91 | 91.7 | 562.5 , 0.855 |
| 2-Ethylfuran † | C6H8O | 96.13 | 81 | 91.9 | 365.0 , 0.810 |
| 2-Pentylfuran * | C9H14O | 138.21 | 81 | 89.7 | 690.5 , 0.690 |
| Heptanal * | C7H14O | 114.19 | 70 | 93.9 | 615.0 , 1.710 |
| Heptane * | C7H16 | 100.20 | 71 | 96.4 | 345.0 , 0.575 |
| Hexadecane | C16H34 | 226.44 | 69 | 87.7 | 965.0 , 0.700 |
| Hexanal * | C6H12O | 100.16 | 149 | 93.4 | 497.5 , 0.695 |
| 2-Ethyl hexanal † | C8H16O | 128.21 | 72 | 84.4 | 680.0 , 0.800 |
| Limonene * | C10H16 | 136.23 | 68 | 91.3 | 732.5 , 0.655 |
| 1-Methyl naphthalene † | C11H10 | 142.20 | 142 | 92.5 | 1042.5 , 1.165 |
| Nonanal * | C9H18O | 142.24 | 57 | 94.7 | 830.0 , 0.795 |
| Octanal * | C8H16O | 128.21 | 57 | 97.2 | 725.0 , 0.735 |
| Octane * | C8H18 | 114.23 | 207 | 91.6 | 460.0 , 0.550 |
| *o*-Cymene † | C10H14 | 134.22 | 119 | 92.5 | 802.5 , 0.690 |
| Pentanal * | C5H10O | 86.13 | 57 | 97.6 | 377.5 , 0.835 |
| Phenol † | C6H6O | 94.11 | 94 | 90.6 | 787.5 , 0.295 |
| 2-Methyl propanal † | C4H8O | 72.11 | 72 | 88.8 | 235.0 , 0.755 |
| *p*-Xylene * | C8H10 | 106.16 | 91 | 95.4 | 570.0 , 0.455 |
| Pyrrole * | C4H5N | 67.09 | 67 | 97.2 | 532.5 , 1.480 |
| Tetradecane * | C14H30 | 198.39 | 57 | 98.1 | 1035.0 , 0.645 |
| Toluene † | C7H8 | 92.14 | 91 | 97.8 | 450.0 , 0.855 |
| Tridecane * | C13H28 | 184.36 | 71 | 88.7 | 975.0 , 0.790 |
| Undecane * | C11H24 | 156.31 | 57 | 87.7 | 782.5 , 0.585 |

| **Metabolite** | **Chr** | **QTL peak (Mbp)** | **Loci** | **Coordinates in bp (5'-3')** | **Enzymes** | **Pathway** |
| --- | --- | --- | --- | --- | --- | --- |
| 2-Acetyl-1-pyrroline, 6M5OTP | 1 | 20.19 | LOC_Os01g37960 | 21,270,839 – 21,265,644 | amidohydrolase |  |
| Pyrrole, 2-Acetyl-pyrrole |  |  | LOC_Os01g38229 | 21,414,995 – 21,441,446 | peptidyl-prolyl isomerase |  |
|  | 2 | 21.9 | LOC_Os02g37090 | 22,426,234 - 22,428,905 | hydrolase, alpha/beta fold family domain containing protein |  |
|  | 6 | 5.34 | LOC_Os06g15990 | 9,091,016 - 9,096,679 | aldehyde dehydrogenase |  |
|  |  |  | LOC_Os06g10420 | 5,356,979 - 5,358,952 | N-carbomoylputrescine amidohydrolase (nitrilase) |  |
|  |  |  | LOC_Os06g10280 | 5,276,590 - 5,285,926 | CDP-alcohol phosphatidyltransferase |  |
|  |  | 27.9 | LOC_Os06g46372 | 28,139,175 – 28,142,555 | dehydrogenase, putative, expressed |  |
|  | 8 | 20.54 | *LOC_Os08g32870 | 20,379,794 – 20,386,061 | aldehyde dehydrogenase |  |
| Heptanal, Octanal, Nonanal, 1-Decene | 1 | 7.92 - 11.16 | LOC_Os01g14080 | 7,882,632 – 7,879,931 | lipase class 3 family protein |  |
|  |  |  | LOC_Os01g14900 | 8,347,893 – 8,351,006 | glycerol-3-phosphate acyltransferase | Glycerolipid metabolism |
|  |  |  | LOC_Os01g15000 | 8,404,247 – 8,408,377 | lipase, putative, expressed |  |
|  |  |  | LOC_Os01g15120 | 8,486,472 – 8,489,700 | hydrolase, alpha/beta fold family domain containing protein |  |
| Heptanal, Octanal, Nonanal | 3 | 12.01 | LOC_Os03g21680 | 12374208 - 12380278 | phospholipid-transporting ATPase |  |
|  |  |  | LOC_Os03g21040 | 11959777 - 11957261 | stress responsive protein, putative, expressed |  |
| 2*E*-Octenal, 2*E*-Nonenal | 6 | 1.5 – 2.03 | LOC_Os06g03830 | 1539028 - 1542212 | oxidoreductase short chain dehydrogenase/reductase family |  |
| 1-Hexanol, 1-Heptanol, 1-Octanol |  |  | LOC_Os06g03890 | 1565577 - 1566225 | alpha-L-fucosidase 3 precursor |  |
| Pentanal, 1-Pentanol |  |  | LOC_Os06g03910 | 1577176 - 1574484 | hydrolase, NUDIX family, domain containing protein |  |
| 2*E*,4*E*-Nonadienal, Hexanal |  |  | LOC_Os06g03990 | 1629717 - 1633629 | aminotransferase, classes I and II, domain containing protein |  |
| 2-Heptanone, 3-Octen-2-one |  |  | LOC_Os06g04420 | 1894306 - 1893929 | putative lipoxygenase |  |
| 2-*n*-Butylfuran, 2-Pentylfuran |  |  |  |  |  |  |

**Table S3.** Genetic regions and putative enzymes associated with volatile compounds and % fatty acid composition in PRD × TMK rice mapping population.

**Table S3.** Continued..

| **Metabolite** | **Chr** | **QTL peak (Mbp)** | **Loci** | **Coordinates in bp (5'-3')** | **Enzymes** | **Pathway** |
| --- | --- | --- | --- | --- | --- | --- |
| Dodecanal | 2 | 11.05 | LOC_Os02g18870.1 | 11009378 - 11014981 | GDSL-like lipase/acylhydrolase, putative, expressed |  |
|  |  |  | LOC_Os02g18954.1 | 11071696 - 11077522 | GDSL-like lipase/acylhydrolase, putative, expressed |  |
|  |  |  | LOC_Os02g18990.1 | 11093810 - 11097793 | GDSL-like lipase/acylhydrolase, putative, expressed |  |
| DMTS, DMDS | 6 | 2.13 | LOC_Os06g04650 | 2030306 - 2028118 | peptide methionine sulfoxide reductase |  |
|  |  |  | LOC_Os06g05690 | 2574177 - 2576722 | cysteine synthase, chloroplast/chromoplast precursor |  |
|  |  |  | LOC_Os06g05700 | 2579088 - 2581726 | cysteine synthase |  |
| DMTS | 5 | 27.84 | LOC_Os05g49070 | 28133217 - 28131776 | dehydrogenase, putative, expressed |  |
|  |  |  | LOC_Os05g50090 | 28708523 - 28705905 | oxidoreductase, 2OG-FeII oxygenase domain containing protein |  |
| C16:1*n*-7, C18:0 | 3 | 29.26 - 34.92 | LOC_Os03g52570 | 30153564 - 30151748 | glycerol-3-phosphate acyltransferase |  |
|  |  |  | LOC_Os03g61720 | 34985196 - 34990759 | glycerol-3-phosphate acyltransferase |  |
| C16:0, C18:3*n*-3 | 6 | 2.026 - 3.72 | LOC_Os06g05550 | 2515760 - 2517940 | GDSL-like lipase/acylhydrolase |  |
|  |  |  | LOC_Os06g04660 | 2030467 - 2034813 | oxidoreductase, 2OG-Fe oxygenase family protein |  |
| C14:0, C16:1*n*-7 | 7 | 21.34 - 23.79 | LOC_Os07g34730 | 20812544–20808033 | acyltransferase |  |
| C18:1*n*-9, C18:2*n*-6 |  |  | LOC_Os07g37580 | 22513093 - 22518756 | diacylglycerol kinase (DGK) |  |
|  |  |  | LOC_Os07g37840 | 22694492 - 22693157 | lipase |  |
|  |  |  | LOC_Os07g39740 | 23816459 - 23814135 | GDSL-like lipase/acylhydrolase |  |
|  |  |  | LOC_Os07g23410 | 13201060-13206929 | omega-6 fatty acid desaturase |  |
